# Multipredictor risk models for predicting individual risk of Alzheimer’s disease

**DOI:** 10.1186/s12967-023-04646-x

**Published:** 2023-10-30

**Authors:** Xiao-He Hou, John Suckling, Xue-Ning Shen, Yong Liu, Chuan-Tao Zuo, Yu-Yuan Huang, Hong-Qi Li, Hui-Fu Wang, Chen-Chen Tan, Mei Cui, Qiang Dong, Lan Tan, Jin-Tai Yu, Alzheimer’s Disease Neuroimaging Initiative

**Affiliations:** 1 Department of Neurology, Qingdao Hospital, University of Health and Rehabilitation Sciences (Qingdao Municipal Hospital), Qingdao, China; 2 Department of Psychiatry, University of Cambridge, Cambridge, United Kingdom; 3 Department of Neurology and Institute of Neurology, WHO Collaborating Center for Research and Training in Neurosciences, Huashan Hospital, Shanghai Medical College, Fudan University, 12th Wulumuqi Zhong Road, Shanghai, 200040 China; 4 School of Artificial Intelligence, Beijing University of Posts and Telecommunications, Beijing, China; 5 PET Center, Huashan Hospital, Fudan University, Shanghai, China

**Keywords:** Alzheimer’s disease, Risk model, Prediction, Cognition

## Abstract

**Background:**

Early prevention of Alzheimer’s disease (AD) is a feasible way to delay AD onset and progression. Information on AD prediction at the individual patient level will be useful in AD prevention. In this study, we aim to develop risk models for predicting AD onset at individual level using optimal set of predictors from multiple features.

**Methods:**

A total of 487 cognitively normal (CN) individuals and 796 mild cognitive impairment (MCI) patients were included from Alzheimer's Disease Neuroimaging Initiative. All the participants were assessed for clinical, cognitive, magnetic resonance imaging and cerebrospinal fluid (CSF) markers and followed for mean periods of 5.6 years for CN individuals and 4.6 years for MCI patients to ascertain progression from CN to incident prodromal stage of AD or from MCI to AD dementia. Least Absolute Shrinkage and Selection Operator Cox regression was applied for predictors selection and model construction.

**Results:**

During the follow-up periods, 139 CN participants had progressed to prodromal AD (CDR ≥ 0.5) and 321 MCI patients had progressed to AD dementia. In the prediction of individual risk of incident prodromal stage of AD in CN individuals, the AUC of the final CN model was 0.81 within 5 years. The final MCI model predicted individual risk of AD dementia in MCI patients with an AUC of 0.92 within 5 years. The models were also associated with longitudinal change of Mini-Mental State Examination (p < 0.001 for CN and MCI models). An Alzheimer’s continuum model was developed which could predict the Alzheimer’s continuum for individuals with normal AD biomarkers within 3 years with high accuracy (AUC = 0.91).

**Conclusions:**

The risk models were able to provide personalized risk for AD onset at each year after evaluation. The models may be useful for better prevention of AD.

**Supplementary Information:**

The online version contains supplementary material available at 10.1186/s12967-023-04646-x.

## Introduction

Alzheimer’s disease (AD) which is the most common cause of dementia in elders, poses a great threat to public health as the size and proportion of the population aged over 65 years continues to increase across the world [1, 2]. AD is thought to have a chronic progressive course that can begin more than 20 years before a clinical diagnosis of dementia can be made [3, 4]. Due to the lack of effective treatments available for AD, early prediction and prevention of AD in individuals with high AD risk has been proposed as a potentially feasible way to delay AD onset and progression [5, 6]. The importance of AD risk prediction has been emphasized for the identification of individuals with high risk of cognitive decline who could benefit from preventive strategies [7, 8].

Increasing numbers of studies have focused on accurate identification of individuals with elevated risk of cognitive decline for early diagnosis and possible intervention. Various risk models have been developed for this purpose. The common variables used in previous reported risk models including demographics, cognitive test scores, lifestyle and health-related variables [9]. Besides, MRI markers, CSF markers and genetic variables were also used in model construction. Previous studies have demonstrated the predictive value of CSF and MRI biomarkers. The models with both MRI and CSF markers might provide predicted risk with higher accuracy. However, the predictive accuracy of many of the existing risk models is only moderate or even low [9]. Furthermore, most of the existing risk models classify patients into different risk categories, and only a few studies on AD prediction at the individual patient level have been reported [10, 11]. Prevention strategies are likely different in individuals with different risks of AD [12]. Precise prediction of AD risk at the individual patient level is needed for employing appropriate prevention strategies. In addition, the new published 2018 NIA-AA research framework indicates that AD and Alzheimer’s pathological changes (without symptoms) are not regarded as separate entities but earlier and later phases of an “Alzheimer’s continuum” [13]. This makes it necessary to construct risk models that predict risk along Alzheimer’s continuum, and not only for a formal diagnosis of Alzheimer Dementia.

In our study, we aimed to construct risk models that best predict incident prodromal stage of AD in cognitive normal individuals and incidence of AD dementia in patients with mild cognitive impairment. We used the model to predict the risks for individuals in each year following initial evaluation, including the estimated years until an individual will be diagnosed with AD. In addition, we aimed to construct a risk model to predict Alzheimer’s continuum (A + T ± N ±) in normal elders (A-T-N-) based on the ATN research framework that diagnoses AD with biomarker evidence of Aβ (A), pathological tau (T), and FDG PET evidence of neurodegeneration or neuronal injury (N).

## Methods

### ADNI dataset

Data used in the preparation for this article were obtained from the Alzheimer’s Disease Neuroimaging Initiative (ADNI) dataset (adni.loni.usc.edu). The ADNI was launched in 2003 as a public–private partnership, led by Principal Investigator Michael W. Weiner, MD. The primary goal of ADNI is to test whether serial magnetic resonance imaging, positron emission tomography, other biological markers, and clinical and neuropsychological assessment can be combined to measure the progression of mild cognitive impairment (MCI) and early AD. For up-to-date information, see www.adni-info.org. The ADNI study was approved by the Institutional Review Board at each of the participating centers, and all participants provided written informed consent.

### Participants

Detailed eligibility criteria of ADNI participants are described at http://www.adni-info.org. Cognitive normal individuals and MCI patients from the ADNI database were included in our study if they were followed-up after 1 year, and subsequently. In brief, cognitive normal (CN) participants had normal cognitive performance (MMSE scores between 24 and 30, Clinical Dementia Rating of 0). Similarly, for participants without MCI or dementia at baseline. MCI patients had MMSE scores between 24 and 30, had objective memory loss measured by the education-adjusted cutoff on the Wechsler Memory Scale Logical Memory II, a CDR of 0.5, and were without dementia.

For Alzheimer’s continuum model construction, individuals from the ADNI were evaluated if they underwent amyloid PET or CSF Aβ analysis (A), CSF p-tau examination (T), and FDG PET (N) at baseline. A cut off value of 1.11 for the florbetapir standardized uptake value ratio (SUVr) and 192 pg/ml for CSF Aβ_42_ were used to determine whether amyloid was abnormal (A +) or normal (A-) [14]. A cutoff value of 23 pg/ml for CSF p-tau level was used to determine whether tau pathology was abnormal (T +) or normal (T-) [14]. And FDG PET (N) (average of angular, temporal, and posterior cingulate) was determined by a cutoff point of 1.21 [15]. to determine abnormal (N +) or normal (N-) neurodegenerative changes. Individuals with normal AD biomarkers (A-T-N-) were included in our study if they were followed-up after 1 year or more. The follow-up period of ATN group was the time between baseline and the final assessment of amyloid PET or CSF Aβ.

### CSF and blood biomarkers measurements

CSF samples were collected at baseline by lumbar puncture. The levels of CSF Aβ, tau, and p-tau were measured by the multiplex xMAP Luminex platform (Luminex Corp., Austin, TX) with Innogenetics (INNOBIA AlzBio3; Ghent, Belgium; for research-use only reagents) immunoassay kit-based reagent. Plasma tau was analyzed with the Human Total Tau kit (research use only grade, Quanterix, Lexington, MA) on the Simoa HD-1 analyzer which uses a combination of monoclonal antibodies for a measure of total tau levels. Plasma NFL level was measured using an in-house ultrasensitive enzyme-linked immunosorbent assay on a single molecule array platform (Quanterix Corp). The assay uses a combination of monoclonal antibodies, and purified bovine NFL as a calibrator. All samples were measured in duplicate.

### Neuroimaging measurements

The magnetic resonance imaging (MRI) measurement protocol in the ADNI dataset has been described in detail elsewhere [16]. In brief, MRI was acquired at multiple sites using a GE Healthcare, Siemens Medical Solutions USA, or Philips Electronics system. Free-surfer software package version 4.3 and 5.1 image processing framework was used to process regional volumes for 1.5 and 3.0 T MRI images, respectively. All the MRI data were reviewed for quality control by the ADNI MRI quality center at the Mayo Clinic. Regional volumes were adjusted for estimated intracranial volume (ICV) (eMethods).

Amyloid PET imaging was measured with florbetapir. The 18F-florbetapir SUVr was calculated by averaging the 18F-florbetapir retention ratio from frontal, anterior cingulate, precuneus, and parietal cortex. The cerebellum was used as a reference region. FDG-PET data were acquired and reconstructed according to a standardized protocol (http://adni.loni.ucla.edu/). Spatial normalization of each individual’s PET image to the standard template was conducted using SPM. For FDG-PET, we averaged counts of angular, temporal, and posterior cingulate regions.

### APOE genotyping and polygenic hazard score computation

The ADNI samples were genotyped with the Omni 2.5 M BeadChip (Illumina, Inc, San Diego, CA) and basic QC was performed. APOE alleles were defined by rs7421 and re429358 which were genotyped by PCR amplification followed by HhaI restriction enzyme digestion and Metaphor Gel. We acquired a Polygenic Hazard Score (PHS) which was computed based on the combination of APOE and 31 other genetic variants from the ADNI database. Detailed information of the PHS can be found in a previous study [17]. In brief, International Genomics of Alzheimer's Project Stage 1 data with genotyped or imputed data was used to identified AD-associated SNPs. Then a PHS score for each participant was provided by a Cox proportional hazard model using genotype data from from Alzheimer's Disease Genetics Consortium phase 1 (excluding individuals from the ADNI).

### Statistical analyses

Both of the CN and MCI groups were separated randomly into discovery and validation cohort comprising 60% and 40% of the original participants respectively, to develop and validate the models. The discovery and validation cohorts of CN group included 292 and 195 samples, respectively. The discovery and validation cohorts of MCI group included 478 and 318 samples, respectively.

The Least Absolute Shrinkage and Selection Operator (LASSO) method was conducted to select significant predictors that influence the time to reach the endpoint during follow-up periods in the discovery cohort with the R package “glmnet”. Using time-to-event data, we conducted LASSO Cox regression for candidate baseline predictors selection and model construction. The possible variables included demographics (age, sex, years of education), risk gene (APOE ε4 status and PHS), health variables (body mass index [BMI], cholesterol level, systolic blood pressure [SBP]), medical history (history of diabetes, hypertension and depression), neuropsychological and functional tests (MMSE, Alzheimer’s Disease Assessment Scale with 11 items [ADAS11], Rey Auditory–Verbal Learning Test [RAVLT] immediate, Functional Assessment Questionnaire [FAQ], Logical Memory Delayed Recall [LM-DR]), neuroimaging markers (white matter hyperintensities [WMH], hippocampus volume, whole brain volume, entorhinal volume, middle temporal lobe volume, ventricles volume), CSF biomarkers (CSF Aβ, tau and p-tau), and blood biomarkers (plasma tau, and neurofilament light protein [NFL]).

Three models were constructed separately for CN and MCI groups. Model 1 were constructed with demographics, neuropsychological tests, health variables and medical history which can be easily available from primary care assessments. In the construction of Model 2, neuroimaging markers and APOE ε4 status were included as possible variables besides the easily available variables used in model 1. All possible predictors (APOE ε4 was excluded because it was included in PHS) were included as candidate variables for the construction of model 3 to reach high accuracy. The variables were selected by LASSO Cox regression in the discovery cohort of the CN or MCI groups by using the minimum criteria (minimized mean-squared error). The details of the predictors inclusion and selection of each model can be seen in Additional file 1: Figs. S1 and S2.

Simplified risk scores were developed using variables from CN and MCI models with continuous variables categorized into groups. Each variable was assigned a score corresponding to the coefficient from Cox regressions. Multivariate imputation by chained equations was applied to impute missing data with missing rates lower than 20% to reduce possible bias due to data incompleteness. Multivariate imputation by chained equations was applied with the R package “MICE”. Detailed information about missing data is provided in Additional file 1.

The endpoint event of MCI patients and CN participants was incident AD dementia and incident prodromal stage of AD, which was indicated by a CDR–global score of 0.5 or greater [18]. The endpoint event of individuals with normal AD biomarkers (A-T-N-) was progression along the Alzheimer’s continuum (A + T ± N ±) including Alzheimer’s pathologic change (A + T-N-), Alzheimer’s disease (A + T + N ±) and Alzheimer’s and concomitant suspected non Alzheimer’s pathologic change (A + T-N +).

Predictive accuracy of the models was quantified by the area under the time-dependent receiver operating characteristic curve (AUC) using survival data [19]. The cumulative/dynamic receiver operating characteristic (ROC) curves and area under curves were calculated with the R package “timeROC”. The Incident/dynamic AUC was calculated with the R package “RisksetROC”. P ≤ 0.05 were considered statistically significant in all analyses.

The predicted risk of incident prodromal stage of AD or AD dementia of each individual was estimated by risk models in the following steps:

1. Calculate the sum of “coefficient × value” for the individual. β_n_ is the regression coefficients in each model determined by LASSO Cox regression. Variable_n_ is the value of each predictor variable.
  $$M\, = \,\left( {\beta_{1} \, \times \,variable_{1} } \right)\, + \,\left( { \, \beta_{2} \, \times \,variable_{2} } \right)\, + \, \cdots \, + \,\left( { \, \beta_{n} \, \times \,variable_{n} } \right)$$
  $$I\, = \,\left( {\beta_{1} \, \times \,variable_{1} } \right)\, + \,\left( {\beta_{2} \, \times \,variable_{2} } \right)\, + \ldots + \,\left( {\beta_{n} \, \times \,variable_{n} } \right)$$
2. Calculate the sum of “coefficient × mean value” across individuals. β_n_ is the regression coefficients in each model determined by LASSO Cox regression. Mean variable_n_ is the mean value of the variable across individuals.
  $$M\, = \,\left( {\beta_{1} \, \times \,mean\,variable_{1} } \right)\, + \,\left( { \, \beta_{2} \, \times \,mean\,variable_{2} } \right)\, + \cdots + \,\left( { \, \beta_{n} \, \times \,mean\,variable_{n} } \right)$$
3. The estimated AD risk at time t is calculated by the following equation, where survival(t) is the survival rate at time t derived from Cox regression models.
  $$Risk_{(t)} \, = \,1\, - \,\left[ {survival_{(t)} } \right]^{exp(I\, - \,M)}$$

## Results

### Demographic and clinical characteristics of included participants

A total of 487 CN individuals and 796 MCI patients were included from ADNI database. The mean follow-up period of the CN group was 5.6 years and the mean follow-up period of the MCI group was 4.6 years. During the follow-up periods, 139 CN participants had progressed to incident prodromal stage of AD (CDR ≥ 0.5) and 321 MCI patients had progressed to AD dementia. The baseline characteristics of CN individuals and MCI patients are summarized in Table 1.

**Table 1:** Baseline characteristics of participants

	CN (n = 487)		MCI (n = 796)		
	ADNI 1	ADNI 2	ADNI 1	ADNI 2	ADNI GO
n	214	273	360	317	119
Number of events	72	67	206	96	19
Follow-up period (years)	5.1 (3.3)	3.8 (1.9)	4.84 (3.1)	3.9 (1.8)	5.3 (2.0)
Age (years)	75.9 (5.1)	73.0 (6.0)	74.8 (7.3)	70.5 (7.3)	71.85 (8.9)
Sex (F/M)	103/111	147/126	129/231	143/174	54/65
Education (years)	16.1 (2.8)	16.6 (2.5)	15.7 (3.0)	15.92.6	15.17 (2.7)
APOE ε4 carriers (0/1/2)	156/54/4	189/78/6	167/150/43	157/123/37	70/41/8
MMSE	29.1 (1.0)	29.0 (1.2)	27.1 (1.8)	28.1 (1.7)	28.3 (1.7)
ADAS11	6.1 (3.0)	5.7 (2.9)	11.42 (4.4)	9.3 (4.6)	7.5 (3.3)
RAVLT immediate	43.7 (9.0)	45.6 (10.3)	31.0 (9.2)	39.7 (11.1)	42.39 (11.1)
FAQ	0.14 (0.6)	0.3 (1.1)	3.8 (4.5)	2.6 (3.8)	1.0 (1.7)
LM-DR	13.0 (3.5)	13.3 (3.1)	3.9 (2.7)	6.2 (3.4)	8.3 (1.8)
Plasma tau (pg/ml)	2.7 (1.6)	NA*	2.9 (1.7)	NA*	NA*
Plasma NFL (pg/ml)	35.1 (22.0)	29.0 (7.8)	42.6 (27.8)	38.3 (17.9)	38.8 (21.3)
White matter hyperintensities (mm^3^)	0.8 (2.2)	6.3 (10.6)	0.8 (2.5)	6.2 (8.3)	7.5 (10.0)
Hippocampus volume (cm^3^)	7.2 (0.9)	7.6 (0.8)	6.4 (1.0)	6.9 (1.1)	7.2 (1.0)
Whole brain volume (cm^3^)	1004.0 (55.5)	1073.4 (64.2)	977.5 (63.9)	1046.4 (64,3)	1069.6 (50.8)
Entorhinal volume (cm^3^)	3.8 (0.7)	3.9 (0.6)	3.3 (0.7)	3.5 (0.7)	3.7 (0.6)
Middle temporal lobe volume (cm^3^)	19.6 (2.1)	21.0 (2.3)	18.5 (2.5)	19.8 (2.4)	20.8 (2.1)
Ventricles volume (cm^3^)	35.2 (16.6)	35.2 (16.6)	41.2 (20.2)	38.2 (18.7)	38.0 (19.3)
CSF Aβ (pg/ml)	207.6 (52.0)	198.2 (49.7)	199.0 (52.1)	175.4 (51.5)	192.2 (48.4)
CSF tau (pg/ml)	69.0 (26.3)	66.2 (32.9)	100.5 (52.2)	98.4 (52.5)	84.2 (58.7)
CSF p-tau (pg/ml)	26.4 (14.2)	35.2 (19.2)	35.82 (22.8)	45.6 (25.1)	34.2 (19.8)
BMI	26.4 (4.5)	27.7 (5.0)	25.99 (4.0)	27.3 (5.2)	28.3 (5.6)
Cholesterol (mg/dl)	193.2 (40.4)	190.9 (39.4)	197.4 (39.2)	208.0 (37.1)	210.4 (39.7)
Systolic blood pressure (mmHg)	132.9 (16.0)	133.8 (16.0)	133.1 (17.0)	130.1 (17.6)	134.8 (18.2)
History of diabetes (%)	13 (6.1%)	25 (9.2%)	24 (6.7%)	33 (10.4%)	12 (10.1%)
History of hypertension (%)	96 (44.9%)	127 (46.5%)	176 (48.9%)	147 (46.4%)	56 (47.1%)
History of depression (%)	35 (16.4%)	59 (21.6%)	103 (28.6%)	118 (37.2%)	29 (24.4%)

Summarized as mean ± standard deviation (SD) for continuous data and count and percentage (%) for categorical data

*ADAS11* Alzheimer’s Disease Assessment Scale with 11 items, *BMI* body mass index, *CSF* cerebrospinal fluid, *FAQ* Functional Assessment Questionnaire, *MMSE* Mini Mental State Examination, *p-tau* phosphorylated Tau, *RAVLT* Rey's Auditory Verbal Learning Test

^*^Data of plasma tau was available from ADNI 1 participants only

Among the individuals who underwent amyloid PET (n = 50) or CSF Aβ (n = 1149) analysis, CSF p-tau (n = 1149) and FDG PET (n = 988) at baseline, a total of 72 participants had normal AD biomarkers (A-T-N-) including those followed-up for a year or more. The ATN group included 33 CN individuals and 39 MCI patients. The mean follow-up period was 3.1 years. During the follow-up periods, 12 subjects progressed along the Alzheimer’s continuum (A + T ± N ±). The baseline characteristics of ATN group are summarized in Additional file 1: Table S1.

### Models for predicting incident prodromal stage of AD in CN participants

**Fig. 1:**
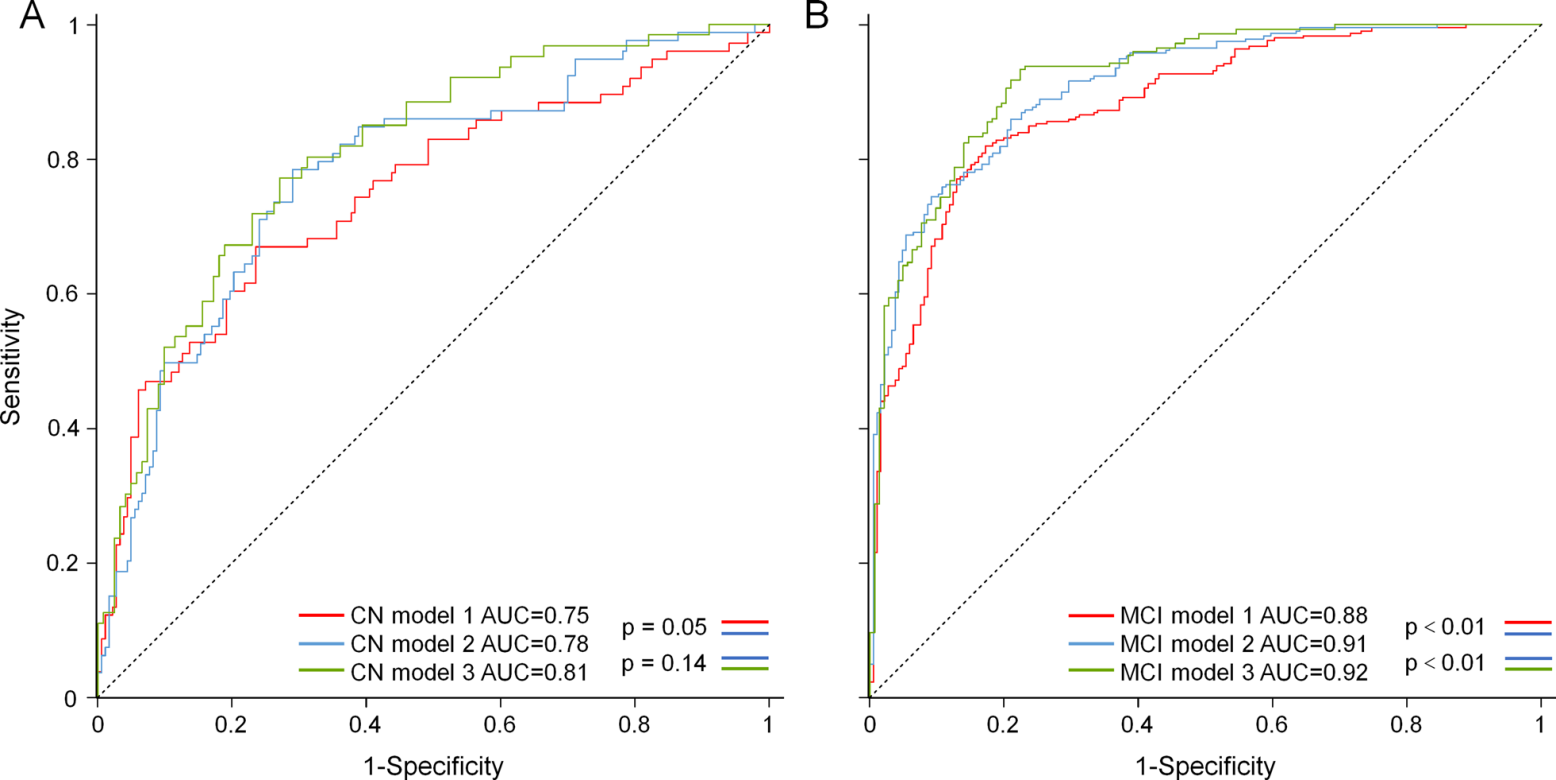
Prediction accuracies of CN and MCI risk models. **A** CN Model 1, Model 2 and Model 3 predicted incident prodromal stage of AD in CN participants within 5 years with AUCs of 0.75 (95% CI 0.69–0.82), 0.78 (95% CI 0.72–0.84) and 0.81 (95% CI 0.74–0.87), respectively. **B** MCI Model 1, Model 2 and Model 3 predicted AD dementia in MCI patients within five years with AUCs of 0.88 (95% CI 0.85–0.91), 0.91 (95% CI 0.88–0.94) and 0.92 (95% CI 0.88–0.95), respectively

CN model 1 included age, SBP, ADAS11, RAVLT, LM-DR, FAQ, history of diabetes and history of depression as variables (CN Model 1, Table 2). The model has an acceptable accuracy for predicting whether an individual will develop incident prodromal stage of AD within 5 years, the AUC was 0.75 (95% CI 0.69–0.82, Fig. 1A) with a specificity of 0.72 (95% CI 0.69–0.75) and a sensitivity of 0.69 (95% CI 0.64–0.74) at the optimal cutoff 0.22. The model had a negative predictive value of 0.91(95% CI 0.90–0.93) and a positive predictive value of 0.35 (95% CI 0.31–0.40). The model showed consistent accuracy in the discovery cohort (AUC 0.75, 95% CI 0.63–0.85, Additional file 1: Fig. S3A) and in the validation cohort (AUC 0.74, 95% CI 0.64–0.84, Additional file 1: Fig. S3B). We also developed a simplified risk score using the variables from CN Model 1 (Additional file 1: Fig. S4).

**Table 2:** Coefficients of each model from lasso regressions

	Model 1	Model 2	Model 3
CN models			
Age	0.0296	0.0162	0.0067
ADAS11	0.0644	0.0579	0.0192
RAVLT	−0.0022	−0.0042	−0.0153
LM-DR	−0.1032	−0.0984	−0.0742
FAQ	0.1939	0.2296	0.2116
History of depression	0.4837	0.5139	0.2621
History of diabetes	0.4864	0.5346	–
Systolic blood pressure	0.0088	0.0107	–
APOE ε4 status	–	0.3461	–
Hippocampus volume	–	−0.2277	−0.2717
CSF Aβ	–	–	−0.0052
CSF p-tau	–	–	0.0140
MCI models			
Age	0.0025	–	
ADAS11	0.0643	0.0674	0.0474
MMSE	−0.0243	−0.0120	−0.0019
RAVLT	−0.0324	−0.0316	−0.0292
LM-DR	−0.1060	−0.0647	−0.0602
FAQ	0.0843	0.0746	0.0797
History of depression	0.0013	0.0020	0.0680
APOE ε4 status	–	0.4064	–
Hippocampus volume	–	−0.0653	−0.1838
Whole brain volume	–	−0.0030	−0.0020
CSF Aβ	–	–	−0.0044
CSF p-tau	–	–	0.0074
Polygenic hazard score	–	–	0.2257
ATN model			
MMSE	0.0097	–	–
APOE ε4 status	0.7533	–	–
Entorhinal volume	−0.6013	–	–
Ventricles volume	0.0059	–	–
CSF Aβ	−0.0488	–	–
CSF tau	0.0162	–	–

After variable selection by LASSO Cox regression, CN Model 2 included age, SBP, ADAS11, RAVLT, LM-DR, FAQ, history of diabetes and history of depression, APOE ε4 status and hippocampus volume (Table 2). The AUC was improved to 0.78 (95% CI 0.72–0.84, Fig. 1A) with the inclusion of new variables with a specificity of 0.74 (95% CI 0.71–0.77) and a sensitivity of 0.74 (95% CI 0.69–0.79) at the optimal cutoff 0.23. The model had a negative predictive value of 0.93 (95% CI 0.91–0.94) and a positive predictive value of 0.38 (95% CI 0.34–0.43). The accuracy in discovery cohort (AUC 0.77, 95% CI 0.67–0.86, Additional file 1: Fig. S3C) and validation cohort (AUC 0.78, 95% CI 0.70–0.81, Additional file 1: Fig. S3D) were similar. The Model 2 had significantly higher AUC than CN model 1 (p = 0.05).

Age, ADAS11, RAVLT, LM-DR, FAQ, history of depression, hippocampus volume, CSF Aβ and CSF p-tau were selected for inclusion in CN Model 3 (Table 2). The AUC of CN Model 3 was 0.81 (95% CI 0.74–0.87, Fig. 1A) with a specificity of 0.81 (95% CI 0.78–0.85) and a sensitivity of 0.67 (95% CI 0.61–0.73) at the optimal cutoff −2.38. The model had a negative predictive value of 0.91(95% CI 0.89–0.93) and a positive predictive value of 0.46 (95% CI 0.39–0.52). CN Model 3 predicted incident prodromal stage of AD within 5 years with high accuracy in both discovery cohort (AUC 0.83, 95% CI 0.75–0.91, Additional file 1: Fig. S3E) and validation cohort (AUC 0.79, 95% CI 0.68–0.89, Additional file 1: Fig. S3F). The difference of AUCs between CN Model 2 and Model 3 was not significant (p = 0.14). A model with CSF Aβ, tau and p-tau as variables was constructed as comparison. The AUC of the model with only CSF biomarkers was 0.67 (95% CI 0.58–0.75) as shown in Additional file 1: Fig. S5.

The performance and stability of the predictive ability of CN risk models for various timeframes was also tested. The time-dependent accuracy measured by Incident/dynamic AUC (iAUC) was stable for the three CN models (Additional file 1: Fig. S6).

A model predicting progression to MCI or dementia in cognitively normal subjects has been reported before [11]. Age, gender, MMSE, CSF Aβ and CSF tau were included in the final model in the study. Harrell’s C-statistic of the model was 0.82. However, the model showed moderate performance (Harrell’s C = 0.62) in the external validation using ADNI data. We also validated their model with our participants. The model predicted prodromal AD within 5 years with an AUC of 0.61 (Additional file 1: Fig. S7).

### Models for predicting AD dementia in MCI participants

MCI model 1 was developed including Age, ADAS11, MMSE, RAVLT, LM-DR, FAQ, History of Depression (Table 2). The model predicted incident AD dementia within 5 years with an AUC of 0.88 (95% CI 0.85–0.91, Fig. 1B) in all the MCI patients and in both discovery (AUC 0.89, 95% CI 0.84–0.93, Additional file 1: Fig. S8A) and validation cohorts (AUC 0.88, 95% CI 0.82–0.93, Additional file 1: Fig. S8B) of MCI patients. The model had a specificity of 0.84 (95% CI 0.81–0.87) and a sensitivity of 0.81 (95% CI 0.78–0.83) at the optimal cutoff −1.24, with a negative predictive value of 0.86 (95% CI 0.84–0.88) and a positive predictive value of 0.77 (95% CI 0.74–0.81). A simplified risk score was developed using variables MCI Model 1 (Additional file 1: Fig. S9).

MCI Model 2 included ADAS11, MMSE, RAVLT, LM-DR, FAQ, History of Depression, APOE ε4 status, hippocampus volume and whole brain volume as variables (Table 2). Compared to MCI Model 1, the AUC of MCI Model 2 was increased to 0.91 (95% CI 0.88–0.94, Fig. 1B) in all MCI patients with a specificity of 0.91 (95% CI 0.89–0.93) and a sensitivity of 0.74 (95% CI 0.72–0.77) at the optimal cutoff -3.77. The model had a negative predictive value of 0.84 (95% CI 0.82–0.86) and a positive predictive value of 0.85 (95% CI 0.82–0.88). MCI Model 2 was also tested in the validation cohort (AUC 0.90, 95% CI 0.86–0.95, Additional file 1: Fig. S8D) with consistent accuracy to that in the discovery cohort (AUC 0.92, 95% CI 0.88–0.95, Additional file 1: Fig. S8C). The AUC of MCI Model 2 was significantly higher than that of MCI model 1 (p < 0.05).

ADAS11, MMSE, RAVLT, LM-DR, FAQ, history of depression, hippocampus volume, whole brain volume, CSF Aβ, CSF p-tau and polygenic hazard score were selected as final variables in MCI Model 3 (APOE ε4 was not included as candidate variables in MCI Model 3 because it was included in PHS). The model showed good accuracy in the discovery cohort (AUC 0.94, 95% CI 0.91–0.97, Additional file 1: Fig. S8E) and in the validation cohort (AUC 0.90, 95% CI 0.85–0.95, Additional file 1: Fig. S8F). The AUC of MCI Model 3 was also significantly higher than that of MCI model 2 (p < 0.05). The model had a specificity of 0.79 (95% CI 0.76–0.82) and a sensitivity of 0.92 (95% CI 0.90–0.94) at the optimal cutoff −4.55, with a negative predictive value of 0.94 (95% CI 0.93–0.96) and a positive predictive value of 0.72 (95% CI 0.68–0.76). A model with CSF Aβ, tau and p-tau as variables was also constructed in MCI participants as comparison. The AUC of the model with only CSF biomarkers was 0.79 (95% CI 0.74–0.84) as shown in Additional file 1: Fig. S10.

MCI Model 1 to Model 3 also showed high stability at various follow-up time points Additional file 1: Fig. S11). The AUCs of MCI Model 1 to Model 3 were 0.77, 0.78 and 0.80, respectively.

Biomarker-based models have been constructed for predicting dementia risk in MCI patients including ADNI participants in a previous study [20]. Their final CSF biomarkers model included age, MMSE, CSF Aβ, CSF tau and hippocampal volume as predictors. The model had adequate prognostic performance with and Harrell’s C of 0.74. Their model was also validated with our participants. The model predicted incident AD dementia within 5 years with an AUC of 0.70 (Additional file 1: Fig. S12).

### Individual risk prediction and association of risk models with longitudinal cognitive decline

The risk of progression to prodromal AD or AD dementia was predicted for every individual with each of the models. Using as an example the MCI model 3, a female MCI patient with no history of depression, with a MMSE score of 28, an ADAS score of 13, a RAVLT score of 35, a LM-DR score of 5, a FAQ score of 2, hippocamppal volume of 8.6 cm^3^, whole brain volume of 1061.8 cm^3^, a CSF Aβ level of 255 pg/ml, a CSF p-tau level of 20.4 pg/ml, and a PHS score of 0.05 had a predicted risk of AD dementia of 1.8% at the first year and 12.3% at the fifth year after initial evaluation. Detailed methods and another illustrative case are shown in Additional file 1: Fig. S13.

**Fig. 2:**
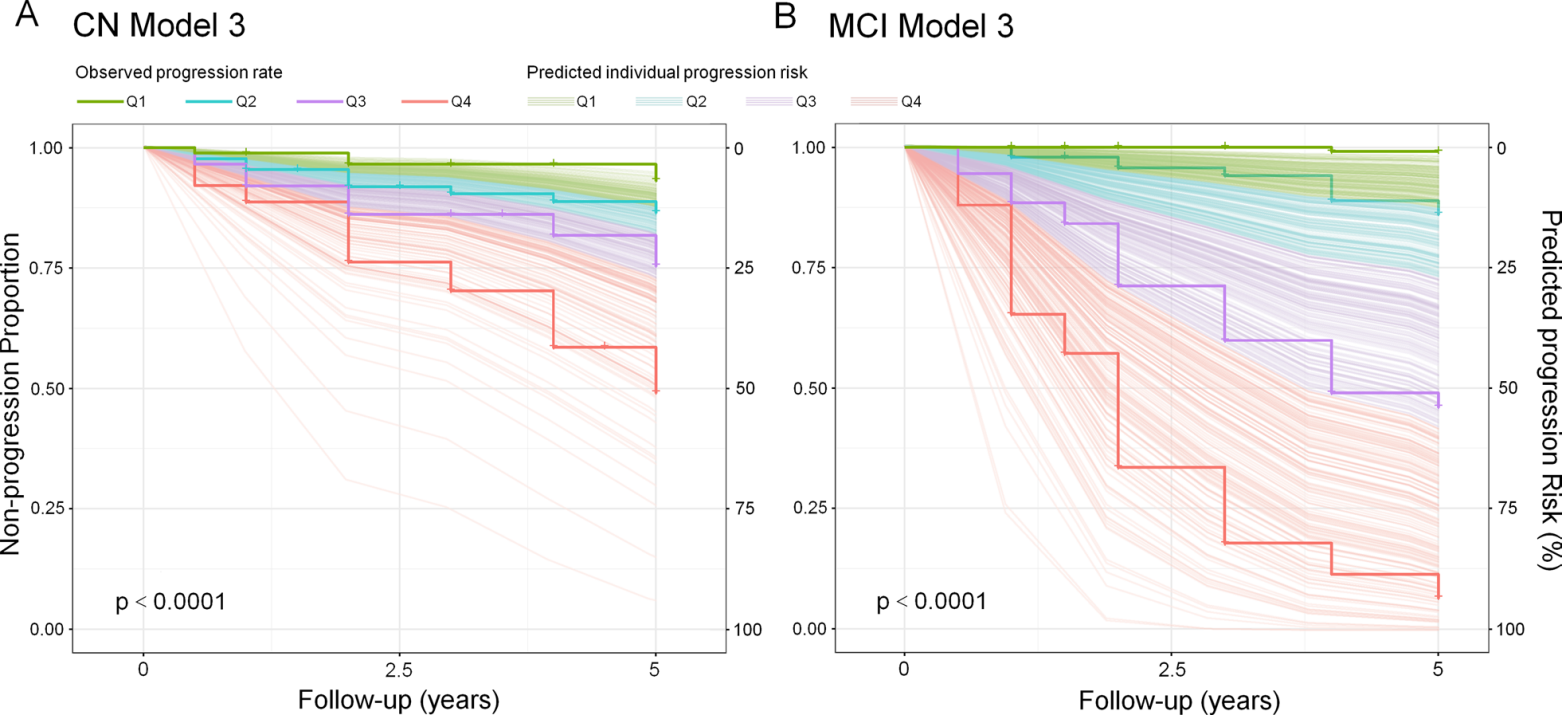
Observed progression rate and trajectories of estimated individual risk of progression. **A** The observed progression rate of prodromal stage of AD analyzed by Kaplan–Meier, was consistent with the individual risk of prodromal AD estimated by CN Model 3. **B** The observed progression rate of AD dementia analyzed by Kaplan–Meier was consistent with the individual risk of AD dementia estimated by MCI Model 3

To compare the predicted individual risk and observed progression rate, we performed Kaplan–Meier analysis. Kaplan–Meier survival curves of CN Model 3 and MCI Model 3 showed that the progression rate of participants in the lowest quartile of the model in follow-up period was much lower than that of participants in the highest quartile in both models (p < 0.0001, Fig. 2A and B). The follow-up individual risk of incident prodromal stage of AD and AD dementia estimated by CN Model 3 and MCI Model 3 is shown as trajectories in Fig. 2A and B. The predicted individual risk shown by longitudinal trajectories appeared to be similar to the observed progression rate shown by Kaplan–Meier survival curves. Predicted risk of incident prodromal stage of AD and AD dementia in each year after onset and estimated years with risk over 20%, 50% and 70% is shown in Additional file 1: Tables S3 and S4.

**Fig. 3:**
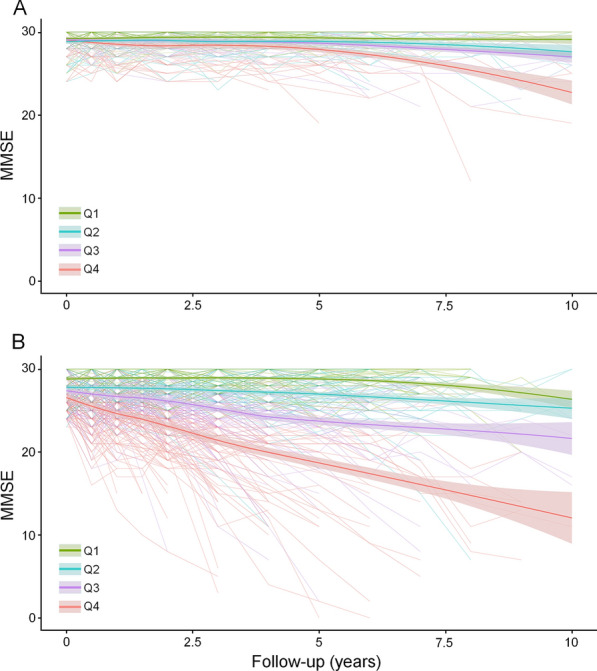
Trajectories of longitudinal MMSE change by quartiles of CN Model 3 and MCI Model 3. **A** The higher quartiles of CN Model 3 showed a higher rate of MMSE decline. **B** The higher quartiles of MCI Model 3 also showed a higher rate of MMSE decline

In addition, the quartiles of CN Model 3 and MCI Model 3 were also associated with a longitudinal change of MMSE (Fig. 3A and B). The same association was also found in other models. Linear regression analyses showed that higher scores from the models were associated with higher rates of decline in MMSE scores (p < 0.001, Additional file 1: Figs. S14 and S15).

### Alzheimer’s continuum model

To predict the initial location on the Alzheimer’s continuum of participants with normal AD biomarkers, the Alzheimer’s continuum model was constructed including MMSE, APOE4 ε4 status, ventricles volume, entorhinal volume, baseline CSF Aβ and baseline CSF tau as variables (Table 2). This model predicted Alzheimer’s continuum (A + T ± N ±) in individuals with normal AD biomarkers (A-T-N-) within 3 years with an AUC of 0.91 (95% CI 0.80–1.00, Additional file 1: Fig. S16).

## Discussion

In this study, we developed risk models using different classes of predictors for accurately predicting risk of progression of Alzheimer’s disease in CN participants and MCI patients at the individual level. In addition, we constructed a risk model for predicting the Alzheimer’s continuum in individuals with normal AD biomarkers, using the 2018 NIA-AA research framework.

Accurate prediction of individuals at high risk of cognitive decline or dementia is important for early intervention, such as monitoring and risk factor-targeted intervention. A model constructed with easily available and low-cost variables like demographics, health factors, cognitive and functional assessments and medical history can be widely used for screening AD risk in primary care settings [21]. CN and MCI Model 1 were developed for this purpose. Both of the models had acceptable accuracy in both the discovery and validation cohorts. For the ease of use of the models, simplified risk scores were generated from CN and MCI models 1. Compared to previously published articles on AD prediction in primary care [21–23], our models could provide information of estimated risk at each year after initial evaluation and the estimated time when an individual’s risk of convert from CN to prodromal stage of AD, or from MCI to AD dementia will increased to certain levels. This could be important in primary care because AD risk provided by the models is straightforward and easily understood by patients.

Poor accuracy has usually been associated with single-factor models in previous studies. Risk models with relatively high accuracy have incorporated multiple factors [12]. Thus, the combination of various risk factors and biomarkers were included in the construction of CN and MCI Models 2 and 3. With all the possible predictors, we applied LASSO Cox regression to select optimal combinations of variables for model construction. Our studies showed that predictive power can be improved by adding Neuroimaging markers, CSF biomarkers and risk genes. The final models reached a high accuracy with AUCs of 0.81 in the prediction of incident prodromal stage of AD in CN participants, and 0.92 in the prediction of AD dementia in MCI patients, which can be considered as good and excellent models, respectively [12]. We also compute tests for comparing the AUCs between models. All the comparisons showed significantly differences except the AUCs between CN model 2 and CN model 3. The AUC of CN model 3 was not significantly higher that of CN model 2. The AUCs should be measured on the same subjects in the comparison of two models. The result might due to the limited number of participants with data of CSF biomarkers. We believed that more available data in the prediction might be optimal in clinical practice.

The NIA-AA research framework was published in 2018 defining AD biologically by neuropathological biomarkers [13]. Multipredictor models that could predict the Alzheimer’s continuum have not been reported in the literature. In this study, an Alzheimer’s continuum model was constructed to predict Alzheimer’s continuum in individuals with normal AD biomarkers. Defining AD as a biological construct might enable a more accurate diagnosis that distinguishes AD from other diseases that could lead to dementia. As well as useful for future AD prediction, this model may also assist the recruitment of individuals with a high risk of AD into clinical trials. The model without CSF biomarkers would be more practical in clinical settings in the prediction of Alzheimer’s continuum. However, the predictive accuracy of the final model without CSF biomarkers as variables was relatively low. Models with only CSF biomarkers as variables were also constructed in both CN and MCI participants. The predictive accuracy of the models was also not high enough (AUC = 0.67 for CN, AUC = 0.79 for MCI). The prediction of prodromal AD and AD dementia with AD biomarkers only was unsatisfactory.

Prediction models were developed before with participants from ADNI using different variables [9]. Gomar et al. have examined the predictive value of different classes of markers including clinical, cognitive, MRI, PET-FDG, and CSF markers in the progression from MCI to AD [24]. They have found that cognitive markers were better predictors than biomarkers. Lehallier et al. also tried to predicted AD in MCI patients using 224 candidate variables [25]. The results of their study suggest that a combination of markers measured in plasma and CSF was useful in predicting of AD dementia. However, none of the reported models using samples from ADNI could provide risk at the individual level. We think individual risk might be more important for patients. In our study, we developed and validated risk models for predicting AD onset at individual level with relatively high accuracy. Risk models that predict individualized risk of progression to dementia have been reported before [10, 11]. Van Maurik et al. have constructed prognostic models for CN and MCI patients based on MRI measures and CSF biomarkers. However, CSF p-tau, which is more important for AD, was not included in their study. Besides, their models for MCI patients can only provide 3- and 1-year progression risks which is relatively short. In our study, we included CSF Aβ, tau and p-tau as candidate variables in model construction. All the candidate variables were selected by LASSO regression. Besides, the risk of prodromal AD or AD dementia at each after evaluation can be predicted with moderate to high accuracy within 5 years.

We also compared our models with existing risk models. A CN model with age, gender, MMSE, CSF Aβ and CSF tau as predictors to predict MCI or AD dementia has been reported by Van Maurik et al. with high accuracy [11]. The Harrell’s C-statistic of the model was 0.82 in their original cohort. The AUC of their model was 0.61 when we validate their model using our participants. The predictive performance of their model was adequate in their own cohort. But the accuracy was low in validation. A MCI model including neuroimaging markers and CSF biomarkers as predictors to predict dementia in MCI patients was constructed in a precious study [20]. Their model showed moderate predictive value in their cohort (Harrell’s C = 0.74) and in our participants (AUC = 0.70). However, the predictive value of their model was relatively low considering the predictors of neuroimaging markers and CSF biomarkers they used in the model construction.

One strength of this study is that individual risks of incident prodromal stage of AD or AD dementia at each year after evaluation, and estimated time when an individual’s risk of convert from CN to incident prodromal stage of AD, or from MCI to AD dementia will increased to certain levels, can be estimated by the models. This might be important for future treatment because intervention strategies for individuals with different risk profiles is likely to be different. With the development of AD prevention strategies, detailed information of future AD risk is necessary for precision prevention. Besides, future decline rate of cognitive function measured by MMSE can also be estimated by the models.

External validation is very important for risk models [26]. The model’s predictions might not be replicable if the model was overfitted. Only a handful of existing model have been externally validated with acceptable accuracy including cardiovascular risk factors, aging, and dementia (CAIDE) models, specifically the Australian National University AD Risk Index (ANU-ADRI) [21, 27–30]. Although participants in this study were all from the ADNI dataset, we separated the entire sample into two cohorts: a discovery cohort and a validation cohort. The models were constructed and validated in the two separate cohorts. The removal of unnecessary variables by LASSO regression also avoided model overfitting.

There are some potential limitations in this study. First, the number of participants is limited, especially those with available data for CSF biomarkers. The diagnosis of AD was recommended by the International Working Group to be restricted to people who have both positive biomarkers and specific AD phenotypes in the newly published article which highlight the importance of biomarkers in the clinical diagnosis of AD [31]. The endpoint used in the construction of CN and MCI models was only clinical due to the small number of individuals with available data for CSF biomarker. The ATN group was not separated into two cohorts for the same reason. Second, the follow-up period was relatively short. The models could only provide predicted risk within 5 years with relatively high accuracy. Further studies with longer follow-up periods may enable long-term predictions. Third, the removal of unnecessary variables was performed by LASSO regression. However, LASSO regression will select one variable from two or more highly collinear variables randomly in the removal of variables. Some important predictors might be removed from the models. Finally, validation of the models with independent cohort is still necessary to test the models’ capabilities and applicability. Future replication studies using independent cohort might be necessary to validate the predictive ability of the models. In future studies, we will perform external validation using our own cohort. More analyses would also be performed to optimize the model with the samples from our cohort.

## Conclusions

In summary, we developed risk models for the prediction from CN to prodromal stage of AD and from MCI to AD dementia using longitudinal data. The risk models were able to provide personalized risk for AD onset at each year after evaluation. Individual risks predicted by the models might be useful for better prevention of AD though external validation of the models with larger samples is still needed.

### Supplementary Information

**Additional file 1: Table S1.** Baseline characteristics of participants with normal AD biomarkers. **Table S2.** Risk of prodromal AD in each year after onset estimated by the risk model in CN subjects. **Table S3.** Risk of AD dementia in each year after onset estimated by the dementia risk model in MCI patients. **Figure S1.** Inclusion and selection of predictors in the construction of CN Models. **Figure S2.** Inclusion and selection of predictors in the construction of MCI Models. **Figure S3.** Prediction of prodromal AD in CN subjects in the discovery population (A, C, E) and in the replication population (B, D, F). **Figure S4.** Risk score developed by the CN risk model 1 for predicting prodromal AD. **Figure S5.** The prediction accuracy of CN risk models with only CSF biomarkers as variables. **Figure S6.** The prediction accuracy of CN risk models at various follow-up time points. **Figure S7.** The prediction accuracy of a previous reported CN model. **Figure S8.** Prediction of AD in MCI patients in the discovery population (A, C, E) and in the replication population (B, D, F). **Figure S9.** Risk score developed by the MCI risk model 1 for predicting Alzheimer’s disease. **Figure S10.** The prediction accuracy of MCI risk models with only CSF biomarkers as variables. **Figure S11.** The prediction accuracy of MCI risk models at various follow-up time points. **Figure S12.** The prediction accuracy of a previous reported MCI model. **Figure S13.** Examples of AD risk calculation with MCI model 3. **Figure S14.** Association of rate of MMSE change with CN risk models. **Figure S15.** Association of rate of MMSE change with MCI risk models. **Figure S16.** Prediction accuracy of Alzheimer’s continuum model.

## Data Availability

The datasets used and/or analyzed during the current study are available from the corresponding author on reasonable request.

## Abbreviations

AD: Alzheimer’s disease
ADAS11: Alzheimer’s disease assessment scale with 11 items
ADNI: Alzheimer’s Disease Neuroimaging Initiative
ANU-ADRI: Australian National University AD Risk Index
AUC: Area under the time-dependent receiver operating characteristic curve
BMI: Body mass index
CAIDE: Cardiovascular risk factors, aging, and dementia
CDR: Clinical dementia rating
CN: Cognitively normal
CSF: Cerebrospinal fluid
FAQ: Functional assessment questionnaire
ICV: Intracranial volume
LASSO: Least absolute shrinkage and selection operator
LM-DR: Logical memory delayed recall
MCI: Mild cognitive impairment
MMSE: Mini Mental State Examination
MRI: Magnetic resonance imaging
NFL: Neurofilament light protein
PHS: Polygenic hazard score
RAVLT: Rey auditory–verbal learning test
ROC: Receiver operating characteristic
SBP: Systolic blood pressure
SUVr: Standardized uptake value ratio
WMH: White matter hyperintensities
