# Supplementary material for: Multipredictor risk models for predicting individual risk of Alzheimer’s disease
Source: J Transl Med. 2023 Oct 30;21:768. doi: 10.1186/s12967-023-04646-x (PMC10614397; doi:10.1186/s12967-023-04646-x)
Supplement: Supplementary file 1 — Additional file 1: Table S1. Baseline characteristics of participants with normal AD biomarkers. Table S2. Risk of prodromal AD in each year after onset estimated by the risk model in CN subjects. Table S3. Risk of AD dementia in each year after onset estimated by the dementia risk model in MCI patients. Figure S1. Inclusion and selection of predictors in the construction of CN Models. Figure S2. Inclusion and selection of predictors in the construction of MCI Models. Figure S3. Prediction of prodromal AD in CN subjects in the discovery population (A, C, E) and in the replication population (B, D, F). Figure S4. Risk score developed by the CN risk model 1 for predicting prodromal AD. Figure S5. The prediction accuracy of CN risk models with only CSF biomarkers as variables. Figure S6. The prediction accuracy of CN risk models at various follow-up time points. Figure S7. The prediction accuracy of a previous reported CN model. Figure S8. Prediction of AD in MCI patients in the discovery population (A, C, E) and in the replication population (B, D, F). Figure S9. Risk score developed by the MCI risk model 1 for predicting Alzheimer’s disease. Figure S10. The prediction accuracy of MCI risk models with only CSF biomarkers as variables. Figure S11. The prediction accuracy of MCI risk models at various follow-up time points. Figure S12. The prediction accuracy of a previous reported MCI model. Figure S13. Examples of AD risk calculation with MCI model 3. Figure S14. Association of rate of MMSE change with CN risk models. Figure S15. Association of rate of MMSE change with MCI risk models. Figure S16. Prediction accuracy of Alzheimer’s continuum model. [file 12967_2023_4646_MOESM1_ESM.docx]

**eMethod:**

**Method for calculating adjusted volume of brain region**

Brain region volumes were adjusted using the linear regression coefficient by regressing intracranial volume (ICV) for each brain region, entered into the following equation:

Adjusted Brain region volume = Raw Brain region volume – β (ICV – Mean ICV)

**Detailed information of missing data**

No data were missing for age, sex, educational levels, APOE4 status, MMSE, ADAS11, RAVLT immediate, FAQ, LM-DR, BMI, cholesterol levels, systolic blood pressure, history of diabetes, history of hypertension and history of depression in both CN and MCI groups. Baseline data were missing for WMH (n=40, 8.2%), hippocamppal volume (n=47, 9.7%), entorhinal volume (n=47, 9.7%), middle temporal lobe volume (n=47, 9.7%), whole brain volume (n=14, 2.9%), ventricles volume (n=25, 5.1%), CSF Aβ (n=134, 27.5%), CSF tau (n=136, 27.9%), CSF p-tau (n=134, 27.5%), plasma NFL (n=38, 7.8%), plasma tau (n=310, 63.7%) and PHS (n=63, 12.9%) in CN group. Baseline data were missing for WMH (n=20, 2.5%), hippocamppal volume (n=121, 15.2%), entorhinal volume (n=123, 15.5%), middle temporal lobe volume (n=123, 15.5%), whole brain volume (n=14, 1.8%), ventricles volume (n=26, 3.3%), CSF Aβ (n=203, 25.5%), CSF tau (n=211, 26.5%), CSF p-tau (n=202, 25.4%), plasma NFL (n=193, 24.2%), plasma tau (n=612, 76.9%) and PHS (n=81, 10.2%) in MCI group.

In individuals with normal AD biomarkers, which included 33 CN individuals and 39 MCI patients, baseline data were missing for WMH (n=4, 5.6%), hippocamppal volume (n=7, 9.7%), entorhinal volume (n=8, 11.1%), middle temporal lobe volume (n=8, 11.1%), whole brain volume (n=1, 1.4%), ventricles volume (n=2, 2.8%), plasma NFL (n=3, 4.2%), plasma tau (n=51, 70.2%) and PHS (n=10, 13.9%)

Multivariate imputation by chained equations was applied to impute missing data with a rate lower than 20% to reduce possible bias due to data incompleteness.

**Detailed statistical methods for the association between risk models and longitudinal rates of MMSE**

Linear mixed models were used to compute the rates of individual longitudinal change of MMSE. The model was adjusted for age, sex, diagnosis and APOE ε4 status. The association between risk models and longitudinal rates of MMSE were analyzed with linear regression models.

Table S1. Baseline characteristics of participants with normal AD biomarkers

|  | ATN group |
| --- | --- |
| n | 72 |
| Number of events* | 12 |
| Follow-up period (years) | 3.1 (1.8) |
| Age (years) | 71.8 (6.8) |
| Sex (F/M) | 41/41 |
| Education (years) | 16.9 (2.5) |
| APOE ε4 carriers (0/1/2) | 65/7/0 |
| MMSE | 28.5 (1.6) |
| ADAS11 | 7.6 (3.7) |
| RAVLT immediate | 41.7 (11.9) |
| FAQ | 1.5 (3.0) |
| LM-DR | 10.0 (4.0) |
| Plasma tau (pg/ml) | 2.7 (0.9) |
| Plasma NFL (pg/ml) | 35.5 (21.0) |
| White matter hyperintensities (mm^3^) | 3.8 (6.3) |
| Hippocampus volume (cm^3^) | 7.5 (1.0) |
| Whole brain volume (cm^3^) | 1052.0 (72.1) |
| Entorhinal volume (cm^3^) | 3.7 (0.6) |
| Middle temporal lobe volume (cm^3^) | 20.5 (2.5) |
| Ventricles volume (cm^3^) | 34.8 (14.9) |
| CSF Aβ (pg/ml) | 234.4 (27.4) |
| CSF tau (pg/ml) | 49.7 (15.9) |
| CSF p-tau (pg/ml) | 18.2 (3.6) |
| BMI | 27.1 (4.8) |
| Cholesterol (mg/dl) | 193.0 (41.0) |
| Systolic blood pressure (mmHg) | 132.1 (16.2) |
| History of diabetes (%) | 7 (9.7%) |
| History of hypertension (%) | 27 (37.5%) |
| History of depression (%) | 27 (37.5%) |

* The events were Alzheimer’s continuum (A+T±N±)

Table S2. Risk of prodromal AD in each year after onset estimated by the risk model in CN subjects

|  | Estimated risk of prodromal AD in each year after onset (%) | | | | | Estimated time with prodromal AD risk over 20% (year) | Estimated time with prodromal AD risk over 50% (year) | Estimated time with prodromal AD risk over 70% (year) |
| --- | --- | --- | --- | --- | --- | --- | --- | --- |
|  | 1 | 2 | 3 | 4 | 5 |  |  |  |
| 1^st^ quartile | <5 | <5 | <5 | 5.7 | 6.7 | >5 | >5 | >5 |
| 2^nd^ quartile | <5 | 7.5 | 9.7 | 11.0 | 13.3 | >5 | >5 | >5 |
| 3^rd^ quartile | 7.2 | 11.5 | 14.3 | 18.0 | 25.4 | 4.0 | >5 | >5 |
| 4^th^ quartile | 12.5 | 22.1 | 31.0 | 40.6 | 50.1 | 1.8 | 4.9 | >5 |

Table S3. Risk of AD dementia in each year after onset estimated by the dementia risk model in MCI patients

|  | Estimated risk of AD in each year after onset (%) | | | | | Estimated time with AD risk over 20% (year) | Estimated time with AD risk over 50% (year) | Estimated time with AD risk over 70% (year) |
| --- | --- | --- | --- | --- | --- | --- | --- | --- |
|  | 1 | 2 | 3 | 4 | 5 |  |  |  |
| 1^st^ quartile | <5 | <5 | <5 | <5 | <5 | >5 | >5 | >5 |
| 2^nd^ quartile | <5 | <5 | 6.7 | 10.3 | 13.9 | >5 | >5 | >5 |
| 3^rd^ quartile | 12.0 | 26.9 | 40.6 | 49.6 | 55.8 | 1.5 | 4.2 | >5 |
| 4^th^ quartile | 31.7 | 62.6 | 83.5 | 89.1 | 94.1 | 0.7 | 1.5 | 2.1 |


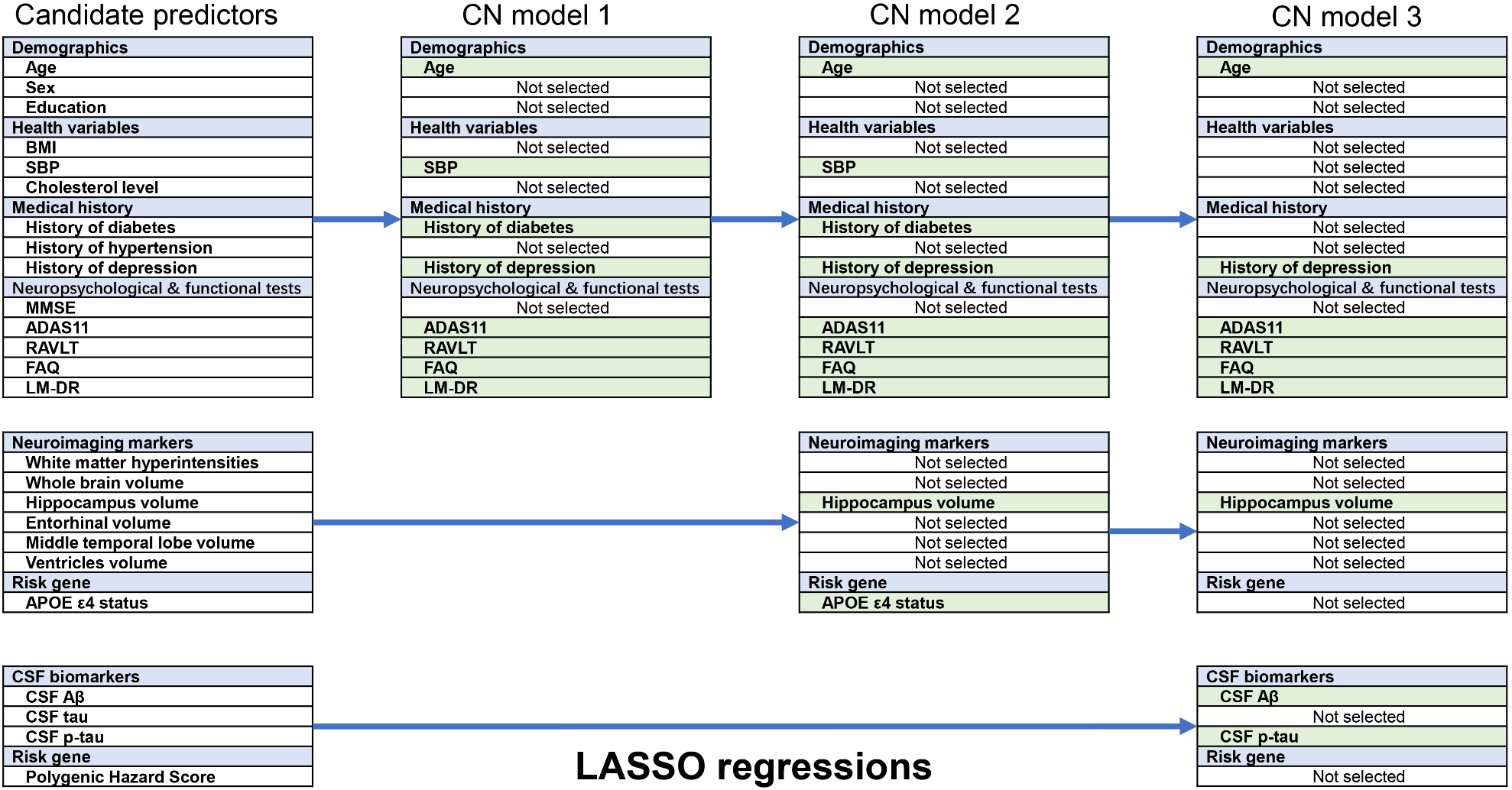


**Figure S1. Inclusion and selection of predictors in the construction of CN Models.**
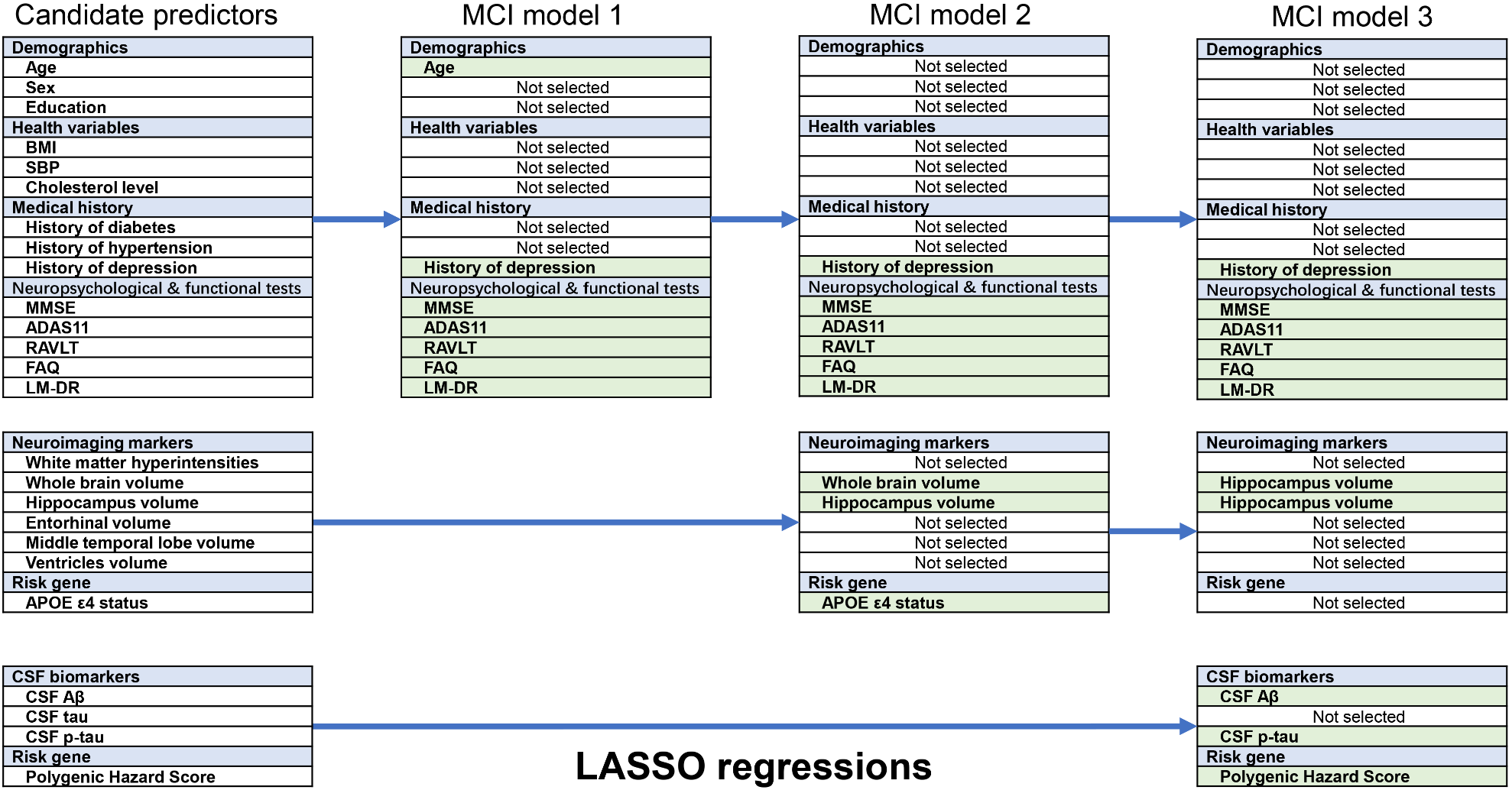


**Figure S2. Inclusion and selection of predictors in the construction of MCI Models.**


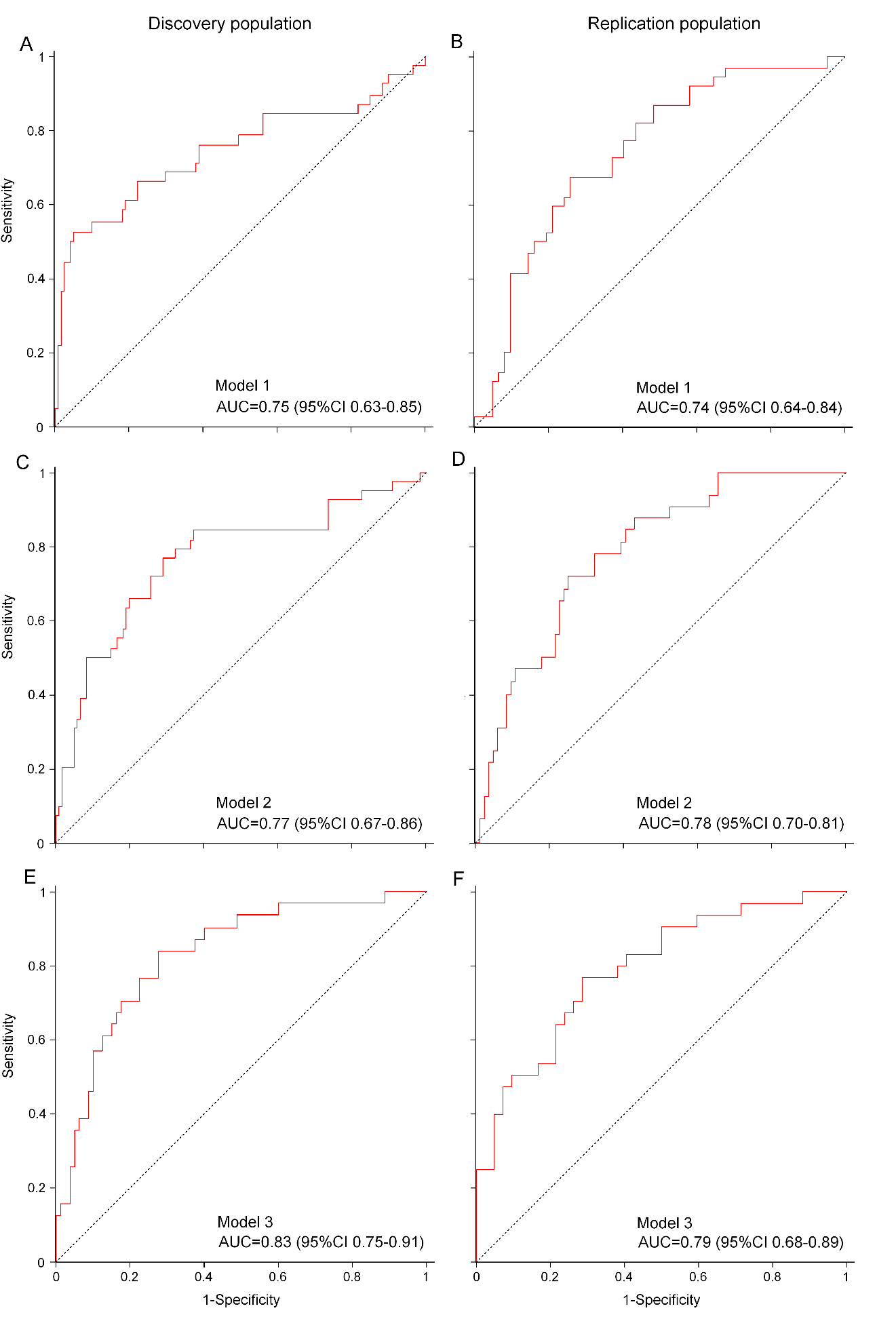


**Figure S3. Prediction of prodromal AD in CN subjects in the discovery population (A, C, E) and in the replication population (B, D, F)**

The CN risk models showed consistent accuracy in discovery population and replication population in CN model 1 (A, B), model 2 (C, D) and model 3 (E, F).


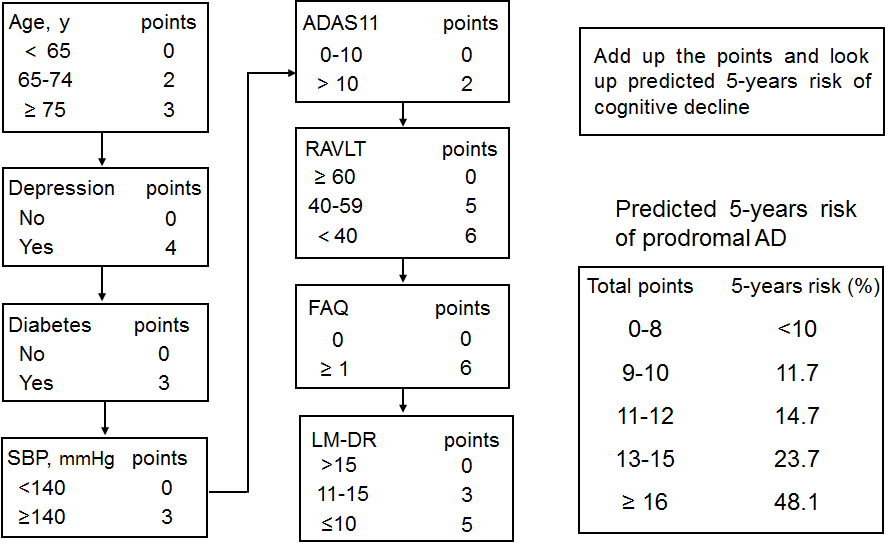


**Figure S4. Risk score developed by the CN risk model 1 for predicting prodromal AD.**

Flow chart of calculating risk of prodromal AD in CN participants using the risk score. Probability of prodromal AD according to the risk score categories was estimated by Kaplan-Meier survival analysis.


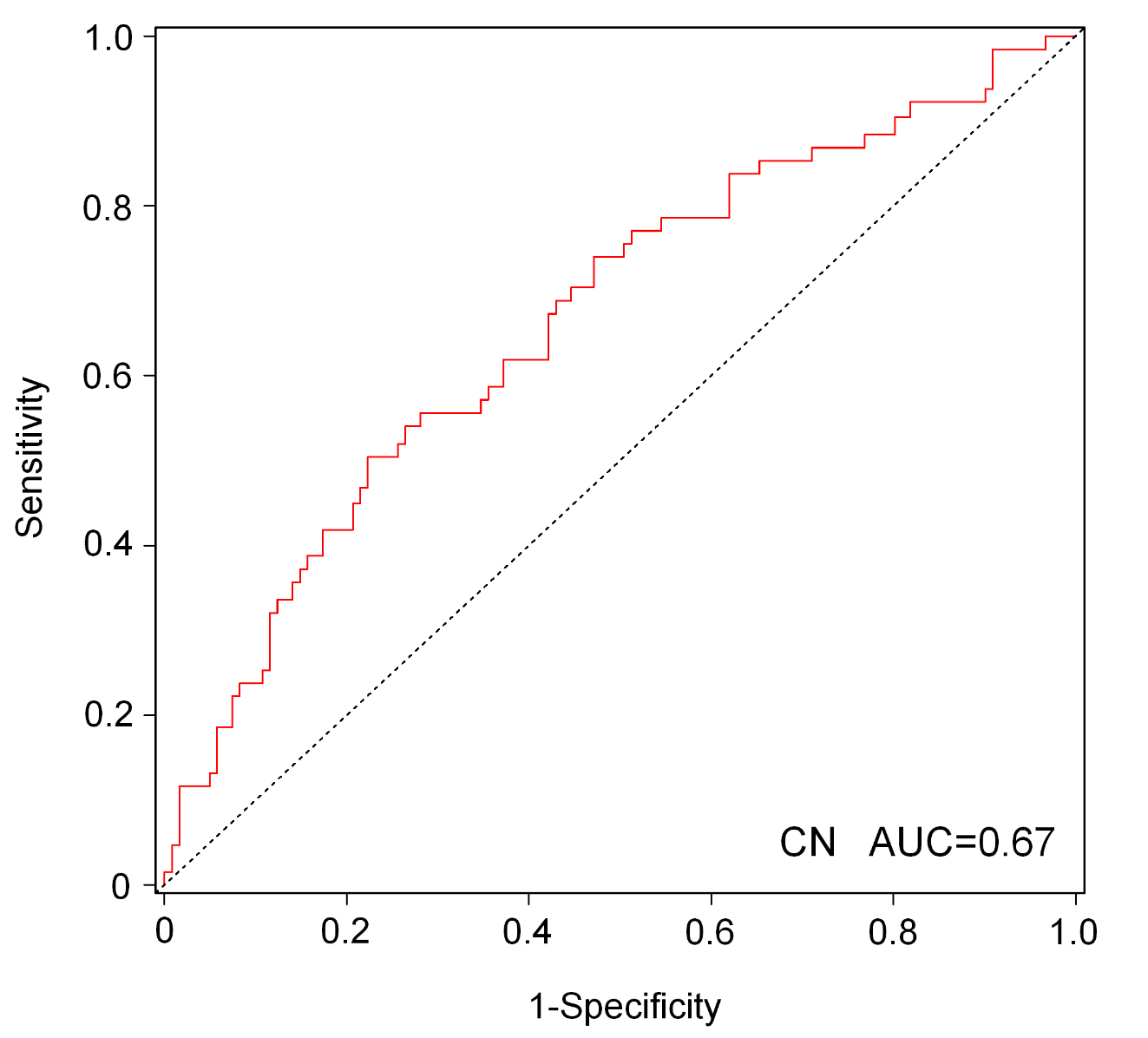


**Figure S5. The prediction accuracy of CN risk models with only CSF biomarkers as variables.**

The CN risk models with only CSF biomarkers as variables predicted incident prodromal stage of AD in CN participants within five years with an AUC of 0.67 (95% CI: 0.58-0.75).


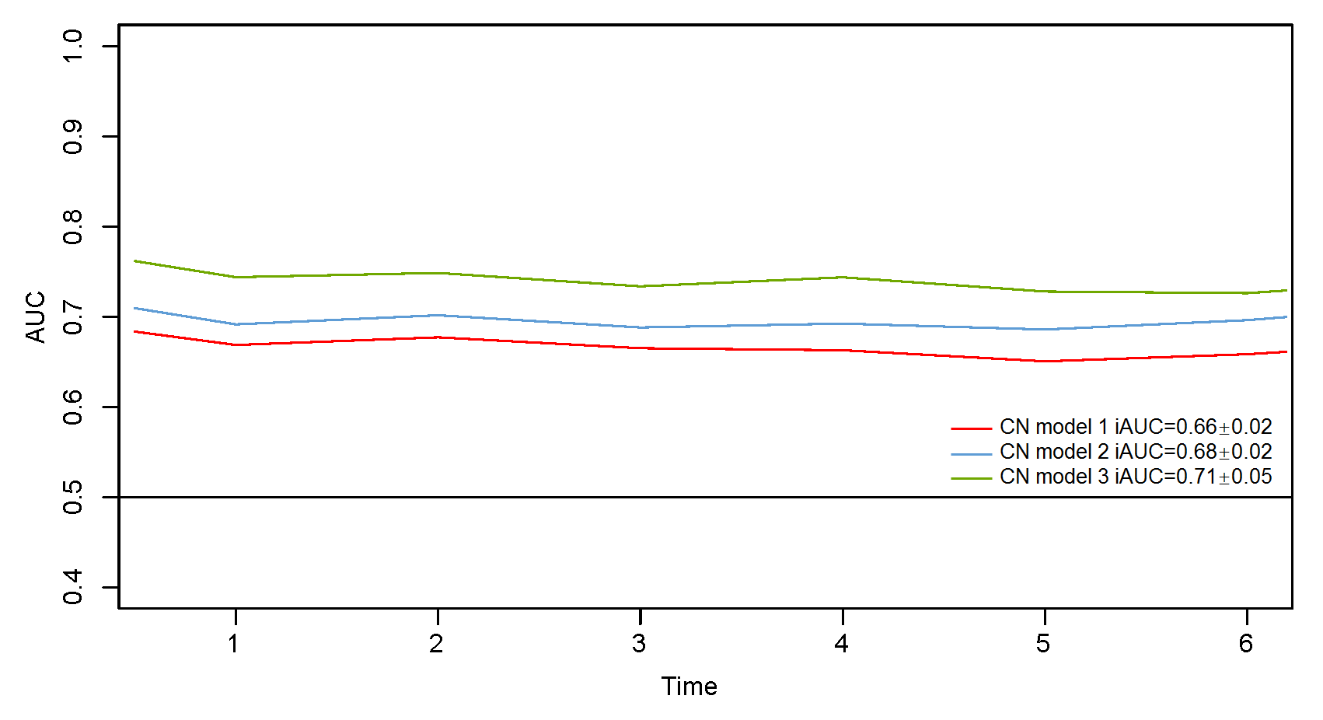
 **Figure S6. The prediction accuracy of CN risk models at various follow-up time points.**

The prediction accuracy was calculated using time-dependent, incident/dynamic ROC curves. The accuracy of all the three CN models was stable up to 5 years since onset.


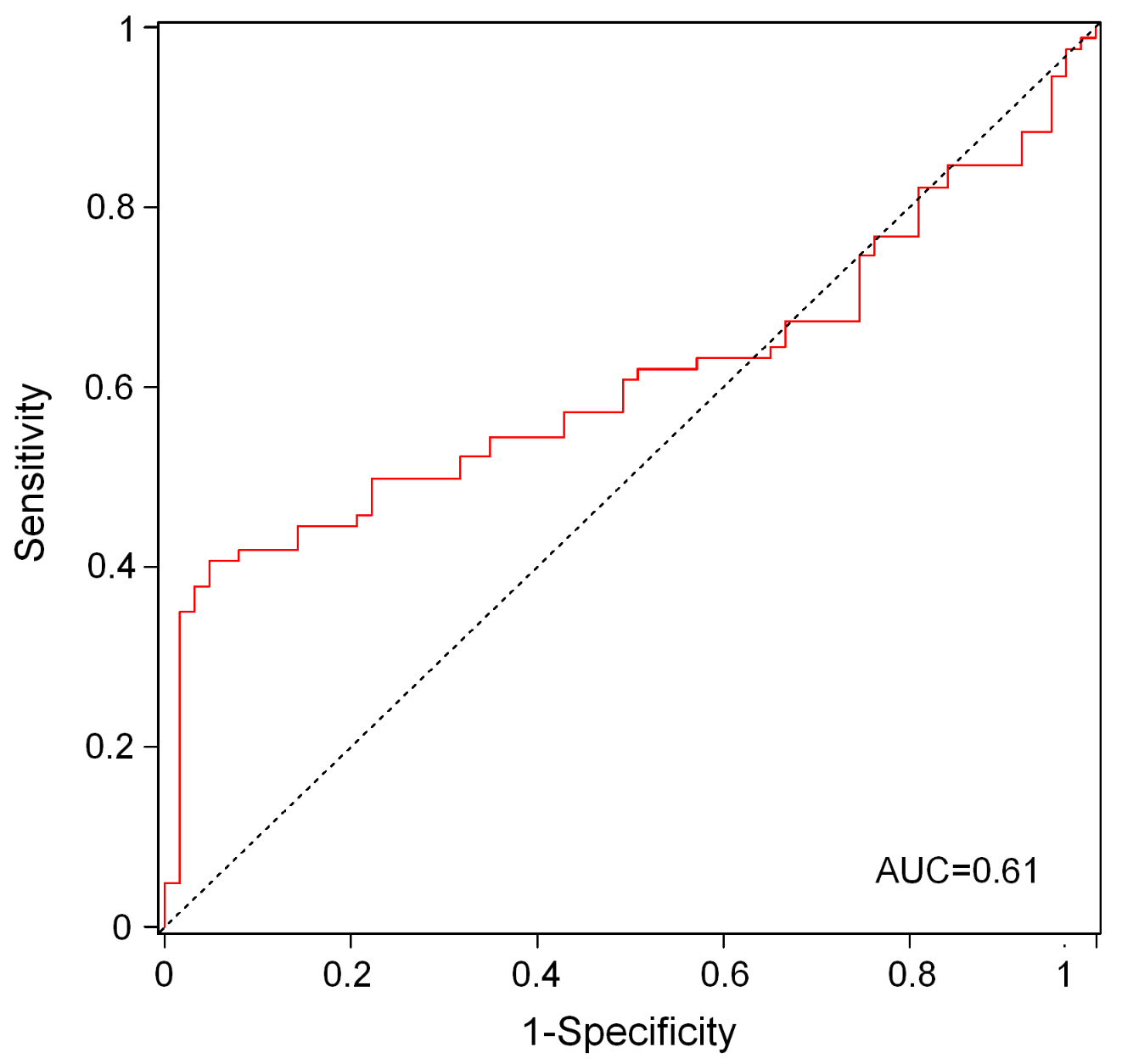


**Figure S7.** **The prediction accuracy of a previous reported CN model.**

The model predicted prodromal AD within five years with an AUC of 0.61.


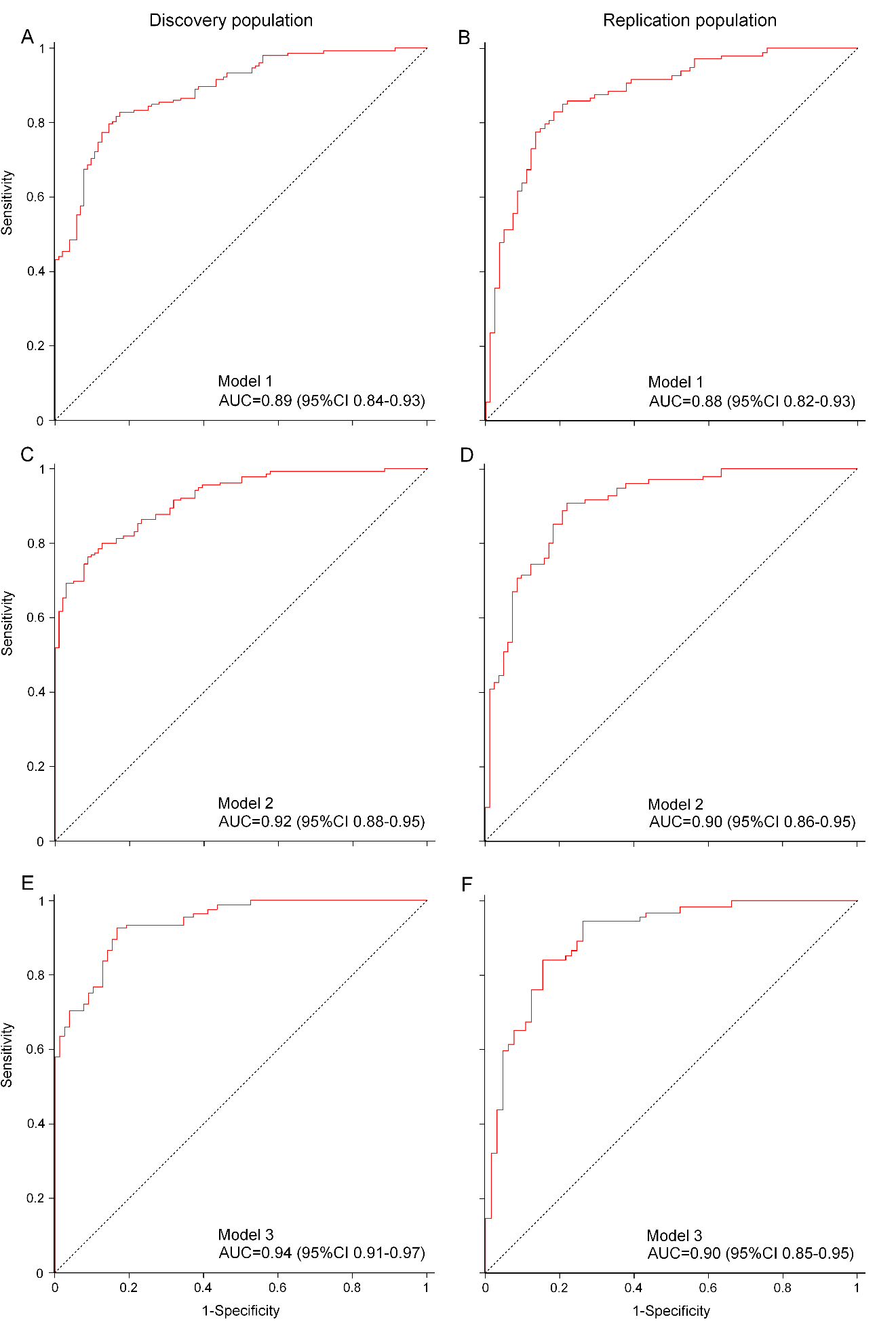


**Figure S8. Prediction of AD in MCI patients in the discovery population (A, C, E) and in the replication population (B, D, F)**

The MCI risk models showed consistent accuracy in discovery population and replication population in MCI model 1 (A, B), model 2 (C, D) and model 3 (E, F).


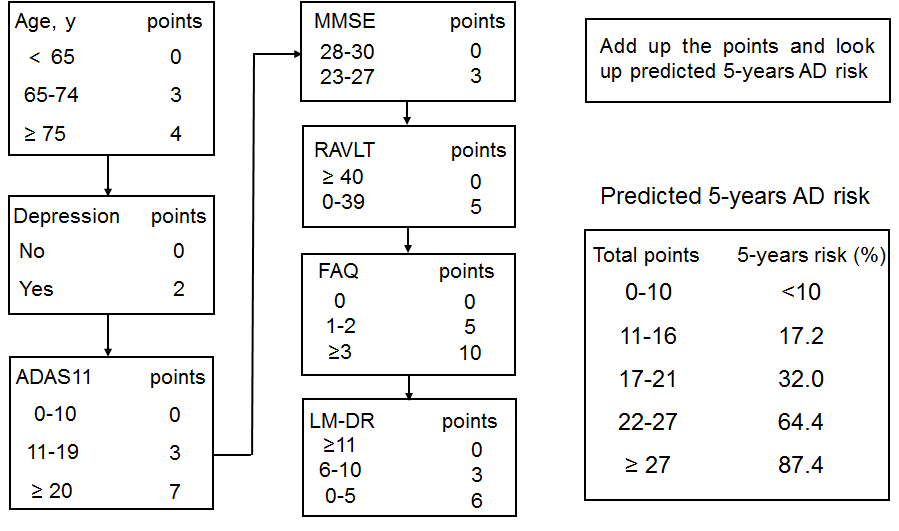


**Figure S9. Risk score developed by the MCI risk model 1 for predicting Alzheimer’s disease.**

Flow chart of calculating risk of AD dementia in MCI patients using the risk score. Probability of AD according to the risk score categories was estimated by Kaplan-Meier survival analysis.


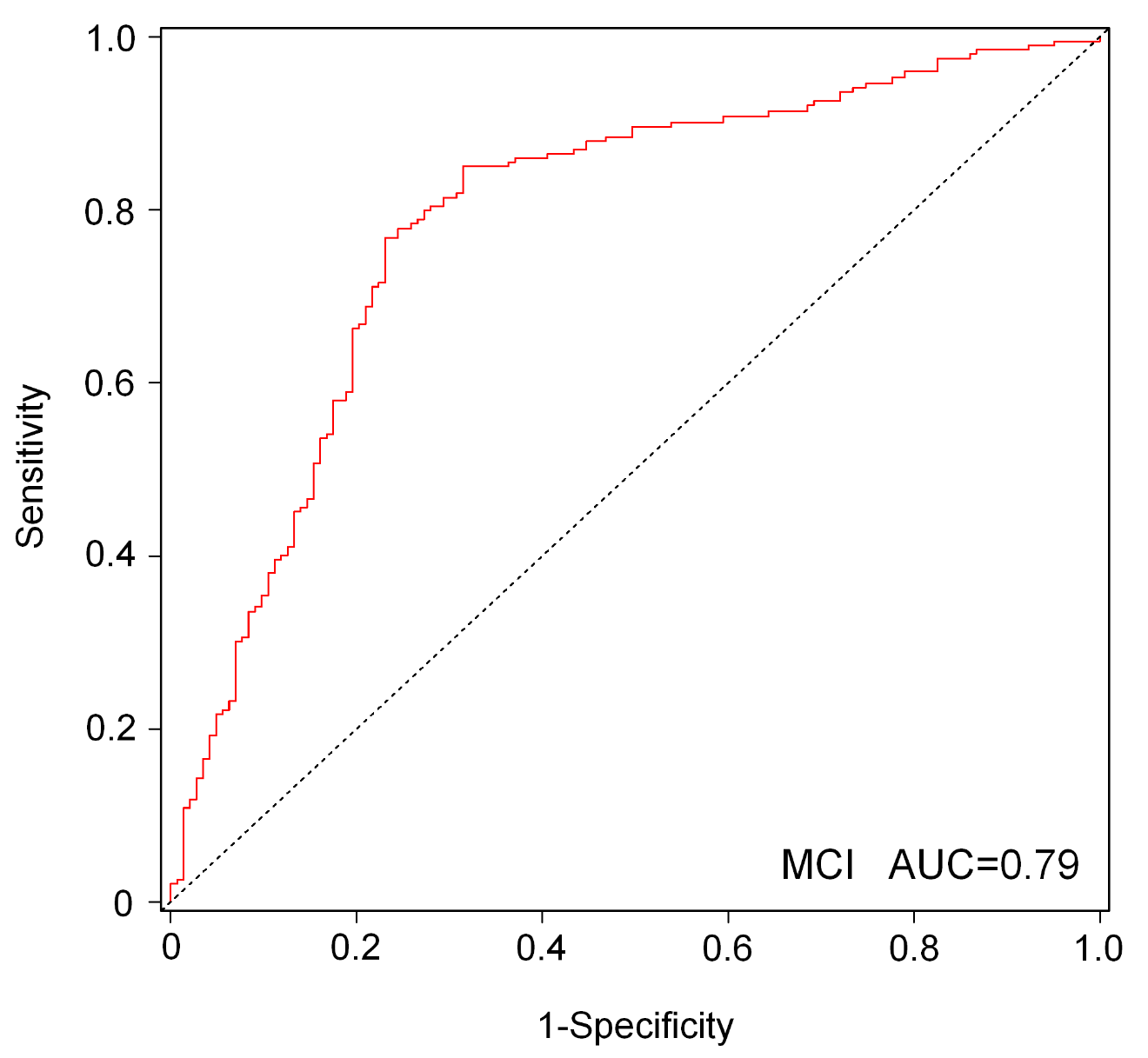
 **Figure S10. The prediction accuracy of MCI risk models with only CSF biomarkers as variables.**

The MCI risk models with only CSF biomarkers as variables predicted incident AD in MCI patients within five years with an AUC of 0.79 (95% CI: 0.74-0.84).


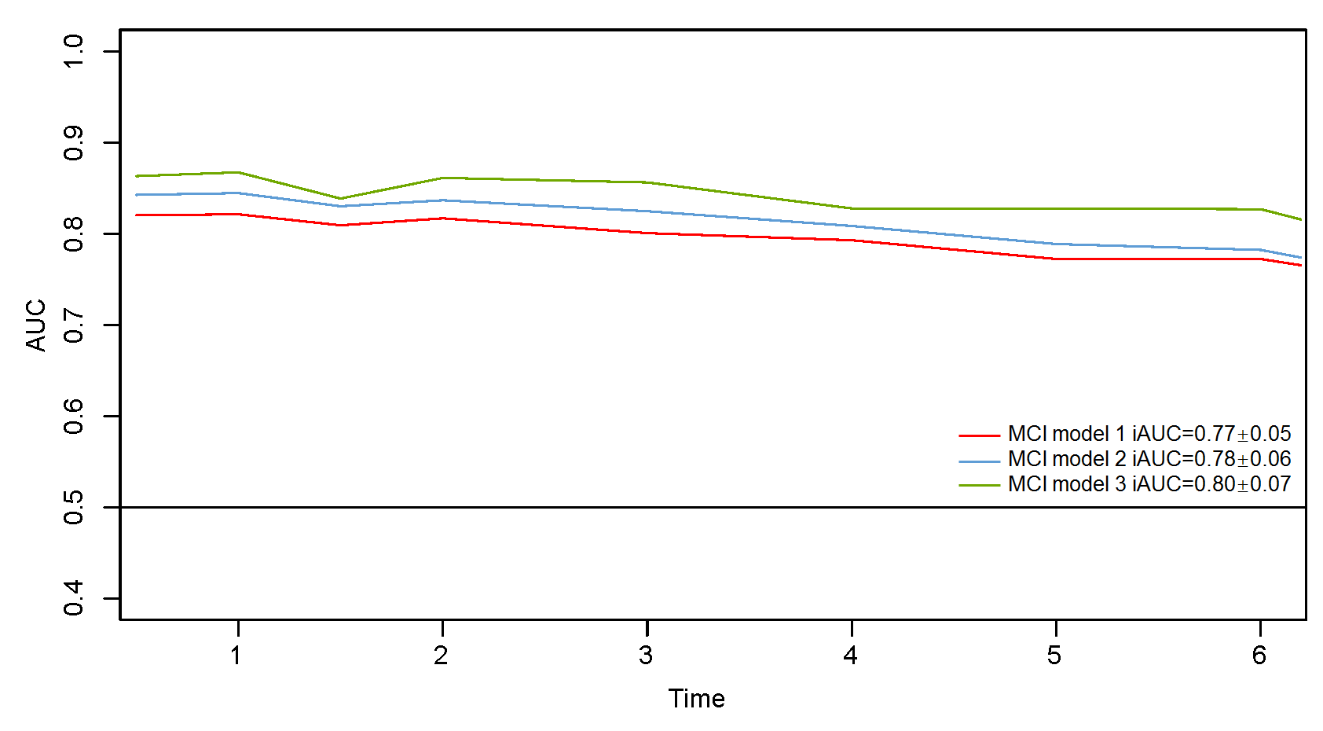
**Figure S11. The prediction accuracy of MCI risk models at various follow-up time points.**

The prediction accuracy was calculated using time-dependent, incident/dynamic ROC curves. The accuracy of all the three MCI models was stable up to 5 years since onset.


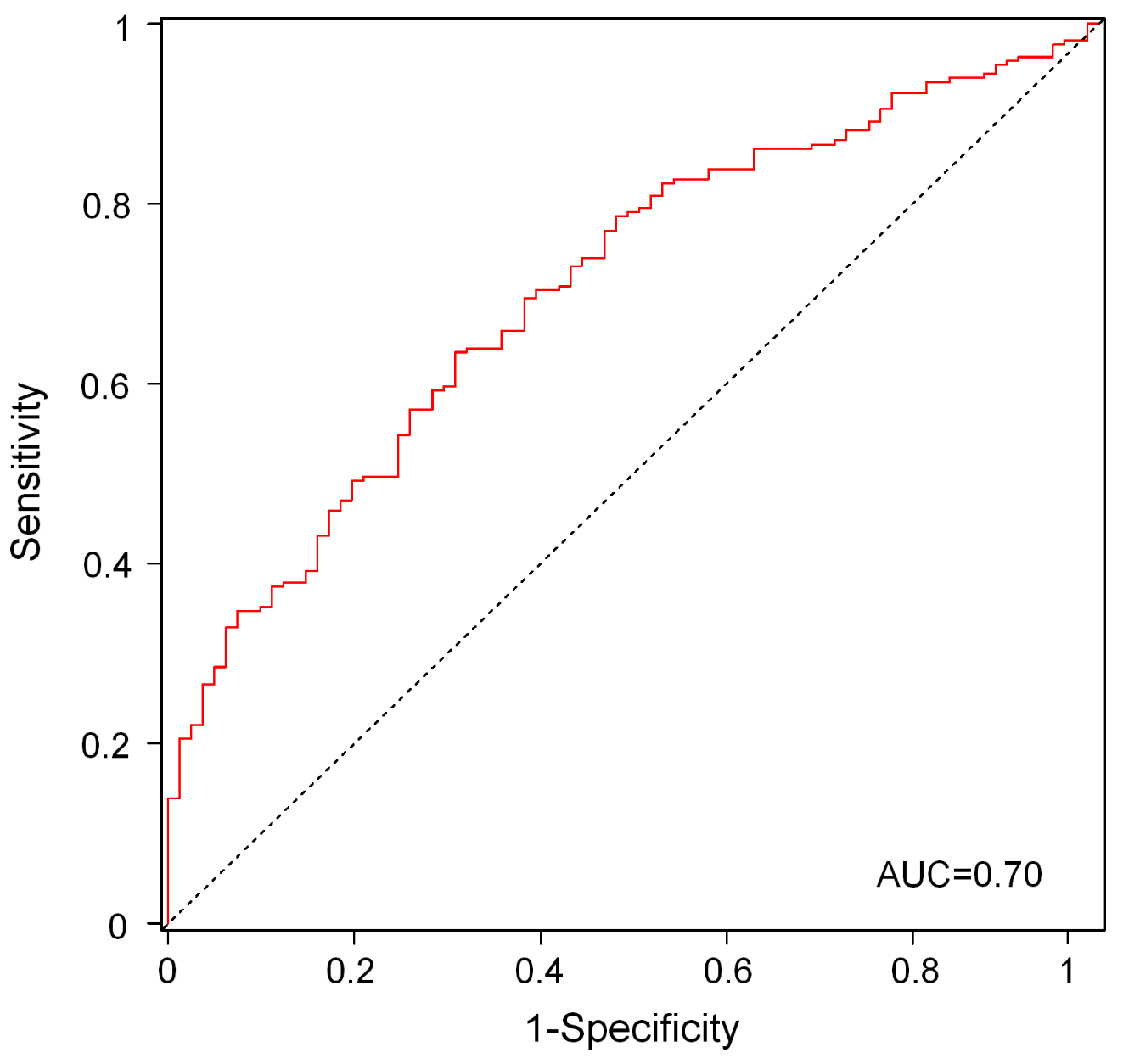


**Figure S12. The prediction accuracy of a previous reported MCI model.**

The model predicted incident AD dementia within five years with an AUC of 0.70.

|  | Coefficient | Ms. A | Mr. B |
| --- | --- | --- | --- |
| ADAS11 | 0.0474 | 13 | 20 |
| MMSE | -0.0019 | 28 | 24 |
| RAVLT | -0.0292 | 35 | 15 |
| LM-DR | -0.0602 | 5 | 5 |
| FAQ | 0.0797 | 2 | 0 |
| History of Depression | 0.0680 | No | No |
| Hippocampus volume | -0.1838 | 8.6 | 6.3 |
| Whole brain volume | -0.0020 | 1061.8 | 1032.6 |
| CSF Aβ | -0.0044 | 255 | 109 |
| CSF p-tau | 0.0074 | 20.4 | 27.8 |
| Polygenic hazard score | 0.2257 | 0.05 | -0.61 |

**
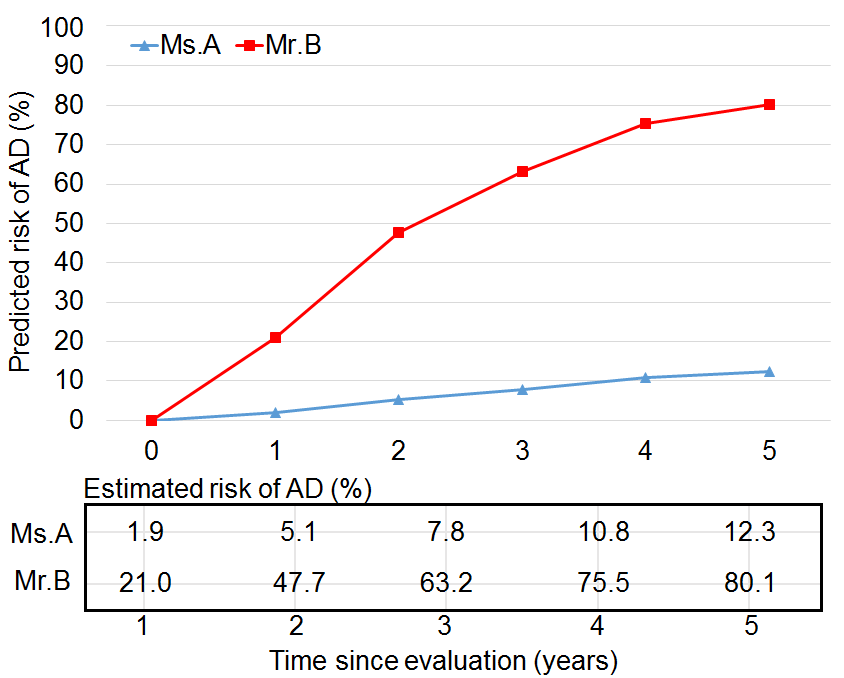
**

**Figure S13. Examples of AD risk calculation with MCI model 3**

A female MCI patient (Ms. A) with no history of depression, with a MMSE score of 28**,** an ADAS score of 13**,** a RAVLT score of 35, a LM-DR score of 5, a FAQ score of 2, hippocamppal volume of 8.6 cm^3^, whole brain volume of 1061.8 cm^3^, a CSF Aβ level of 255 pg/ml, a CSF p-tau level of 20.4 pg/ml, and a PHS score of 0.05 had a predicted risk of AD dementia of 1.8% at the first year and 12.3% at the fifth year after evaluation.

A male MCI patient (Mr. B) with no history of depression with a MMSE score of 20**,** an ADAS score of 24, a RAVLT score of 15, a LM-DR score of 5, a FAQ score of 0, hippocamppal volume of 6.3 cm^3^, whole brain volume of 1032.6 cm^3^, a CSF Aβ level of 109 pg/ml, a CSF p-tau level of 27.8 pg/ml, and a PHS score of -0.61 had an predicted risk of AD dementia of 21.0% at the first year and 80.1% at the fifth year after evaluation.


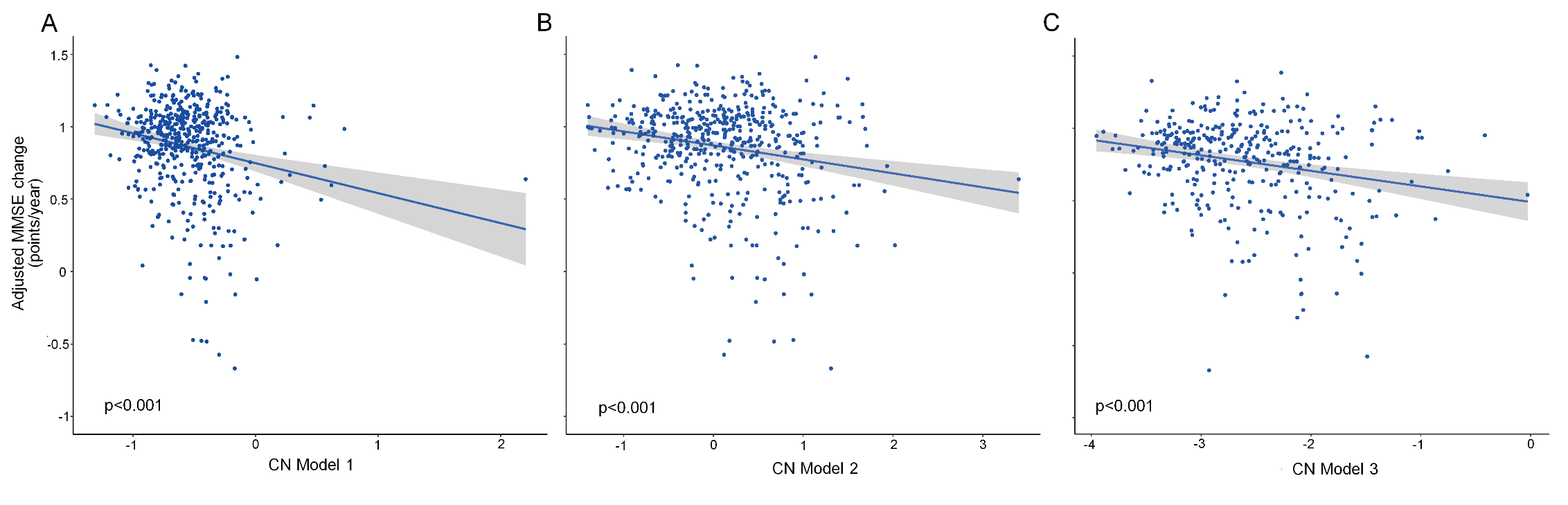


**Figure S14. Association of rate of MMSE change with CN risk models.**

Higher scores from CN risk models were associated with higher rates of decline in MMSE scores in the three CN risk models.


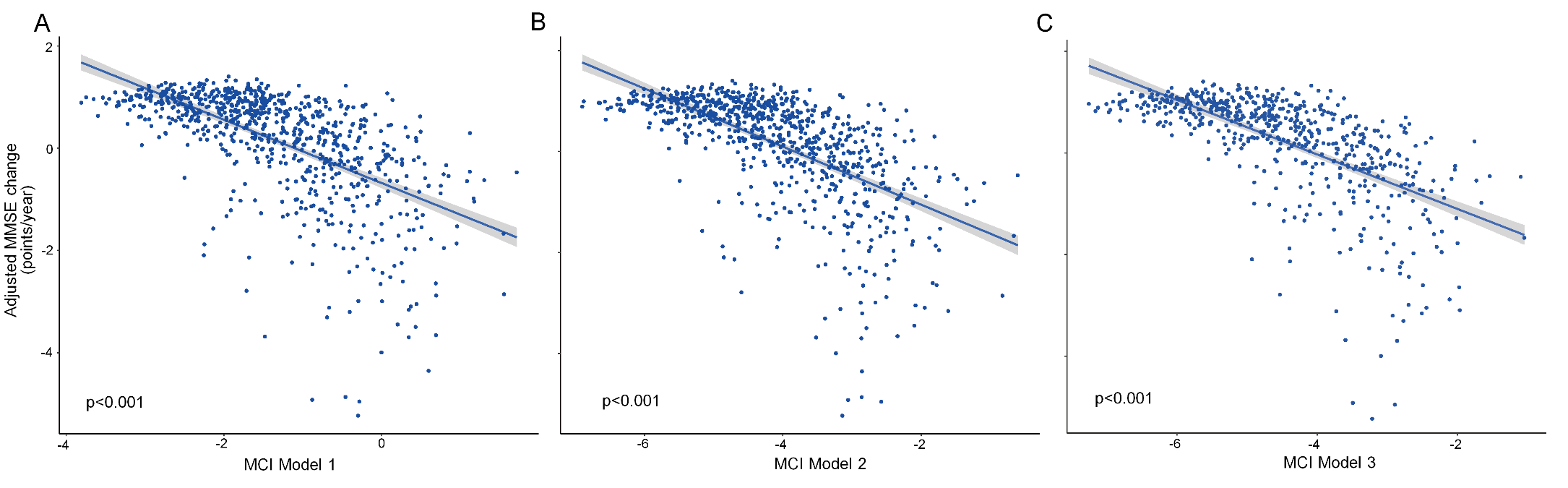


**Figure S15. Association of rate of MMSE change with MCI risk models.**

Higher scores from MCI risk models were associated with higher rates of decline in MMSE scores in the three MCI risk models.


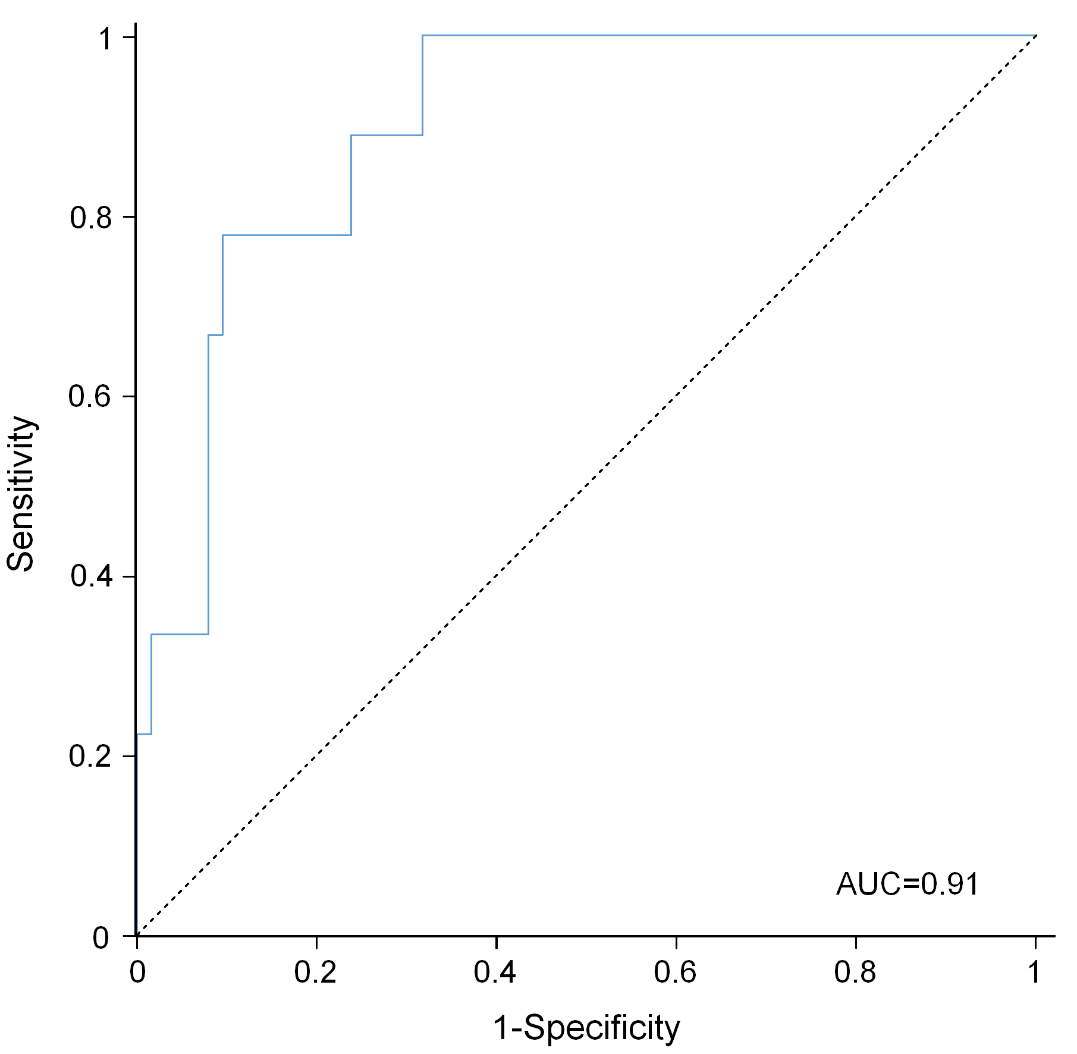


**Figure S16. Prediction accuracy of Alzheimer’s continuum model.**

The Alzheimer’s continuum model predicted Alzheimer’s continuum in individuals with normal AD biomarkers within three years with an AUC of 0.91(95% CI: 0.80-1.00).
